# A molecular module improves rice grain quality and yield at high temperatures

**DOI:** 10.1093/nsr/nwae416

**Published:** 2024-11-26

**Authors:** Feifei Lu, Guiai Jiao, Jiehua Qiu, Shaolu Zhao, Fengli Zhao, Ping Wang, Luna Chen, Pengfei Chen, Xinwei Li, Nannan Dong, Ruijie Cao, Xiaoxue Li, Zheyan Ruan, Gaoneng Shao, Shikai Hu, Zhonghua Sheng, Lihong Xie, Shaoqing Tang, Peisong Hu, Xiangjin Wei

**Affiliations:** State Key Laboratory of Rice Biology and Breeding, China National Center for Rice Improvement, China National Rice Research Institute, Hangzhou 310006, China; State Key Laboratory of Rice Biology and Breeding, China National Center for Rice Improvement, China National Rice Research Institute, Hangzhou 310006, China; State Key Laboratory of Rice Biology and Breeding, China National Center for Rice Improvement, China National Rice Research Institute, Hangzhou 310006, China; State Key Laboratory of Rice Biology and Breeding, China National Center for Rice Improvement, China National Rice Research Institute, Hangzhou 310006, China; State Key Laboratory of Rice Biology and Breeding, China National Center for Rice Improvement, China National Rice Research Institute, Hangzhou 310006, China; State Key Laboratory of Rice Biology and Breeding, China National Center for Rice Improvement, China National Rice Research Institute, Hangzhou 310006, China; State Key Laboratory of Rice Biology and Breeding, China National Center for Rice Improvement, China National Rice Research Institute, Hangzhou 310006, China; State Key Laboratory of Rice Biology and Breeding, China National Center for Rice Improvement, China National Rice Research Institute, Hangzhou 310006, China; State Key Laboratory of Rice Biology and Breeding, China National Center for Rice Improvement, China National Rice Research Institute, Hangzhou 310006, China; State Key Laboratory of Rice Biology and Breeding, China National Center for Rice Improvement, China National Rice Research Institute, Hangzhou 310006, China; State Key Laboratory of Rice Biology and Breeding, China National Center for Rice Improvement, China National Rice Research Institute, Hangzhou 310006, China; State Key Laboratory of Rice Biology and Breeding, China National Center for Rice Improvement, China National Rice Research Institute, Hangzhou 310006, China; State Key Laboratory of Rice Biology and Breeding, China National Center for Rice Improvement, China National Rice Research Institute, Hangzhou 310006, China; State Key Laboratory of Rice Biology and Breeding, China National Center for Rice Improvement, China National Rice Research Institute, Hangzhou 310006, China; State Key Laboratory of Rice Biology and Breeding, China National Center for Rice Improvement, China National Rice Research Institute, Hangzhou 310006, China; State Key Laboratory of Rice Biology and Breeding, China National Center for Rice Improvement, China National Rice Research Institute, Hangzhou 310006, China; State Key Laboratory of Rice Biology and Breeding, China National Center for Rice Improvement, China National Rice Research Institute, Hangzhou 310006, China; State Key Laboratory of Rice Biology and Breeding, China National Center for Rice Improvement, China National Rice Research Institute, Hangzhou 310006, China; State Key Laboratory of Rice Biology and Breeding, China National Center for Rice Improvement, China National Rice Research Institute, Hangzhou 310006, China; State Key Laboratory of Rice Biology and Breeding, China National Center for Rice Improvement, China National Rice Research Institute, Hangzhou 310006, China

**Keywords:** high-temperature stress, grain quality and yield, starch biosynthesis, heat shock protein, rice

## Abstract

Excessive temperatures during grain filling can compromise endosperm starch biosynthesis and decrease grain quality and yield in rice. However, the molecular mechanisms underlying these remain unclear. Here, we show that heat shock protein OsHsp40-1 interacts with and elevates the ATPase activity of OsHsp70-2 in rice. OsHsp40-1 also interacts with the key starch biosynthetic enzymes OsGBSSI and OsPPDKB and thereby enhances their stability and activity, which is essential for maintaining rice quality and grain yield under moderate high-temperature (HT) conditions. Overexpression of *OsHsp70-2* and *OsHsp40-1* in rice significantly improved grain quality and yield at HT. Furthermore, a haplotype analysis identified favorable alleles of *OsHsp70-2* and *OsHsp40-1*, which could be used for improving thermotolerance in rice. Collectively, our findings reveal a novel mechanism by which the OsHsp70-2–OsHsp40-1 module ameliorates the effects of HT on starch biosynthesis, providing a new strategy for genetic improvement of rice quality and yield under HT conditions.

## INTRODUCTION

Global warming is disrupting agricultural production and threatens food security worldwide. For every 1°C increase in global average temperature, the yields of the world's major crops are predicted to decrease by 3% –8% [1]. Rice (*Oryza sativa* L.) is a staple food crop for over half the world's population. Excessive temperatures can perturb multiple physiological processes in rice, including cell membrane integrity, reactive oxygen species (ROS) homeostasis, photosynthesis, carbohydrate synthesis and allocation, and hormonal regulation, ultimately reducing seed set, biomass, and grain quality and yield [2]. Therefore, elucidating the molecular mechanisms of rice responses to heat stress and identifying high-temperature (HT) tolerance genes will have theoretical and practical significance for cultivating HT-resistant rice varieties to ensure food security.

As a heat-adapted crop originating from low latitudes, rice is generally more heat tolerant than other cereal crops such as wheat, and has evolved a variety of mechanisms that enable it to tolerate elevated ambient temperatures [3]. Under HT, an intracellular heat-stress response-signal cascade is triggered that involves the plasma membrane cyclic nucleotide-gated Ca^2+^ ion channels [4,5]. TT3.1 is a potential thermosensor that can interact with and mediate the degradation of TT3.2, thereby enhancing rice thermotolerance and reducing grain-yield losses caused by heat stress [6]. However, previous studies have focused on the effects of HT at the seedling, booting and flowering stages of rice development, with little attention paid to the grain-filling stage. HT at the grain-filling stage usually leads to insufficient accumulation of grain storage compounds and serious chalkiness, in turn causing poor quality and lower yield [4–6]. The molecular mechanisms underlying the negative impacts of HT stress on rice quality and yield, and how rice protects itself against heat stress during the filling stage, remain largely unknown.

Heat shock proteins (HSPs, including HSP40, HSP60, HSP70, HSP90, HSP100 and small HSPs), are a class of proteins upregulated by HT that are also involved in plant responses to a variety of stresses, such as heat, drought, low temperature, excess salt, heavy metals and hormones [7]. For example, OsHSP60-3B interacts with FLO6 to regulate the biosynthesis of starch grains in rice pollen and reduce ROS levels in anthers, thus ensuring the normal development of male gametophytes at HT [8]. The rice mutants of *Hsp70cp-2* (*FLO11*) showed insufficient grain starch synthesis and extremely chalky grains under HT [9,10]. OsHsp101 can interact with OsHsp70cp-2 and starch-related enzymes AGPL1, AGPL3 and PHO1 to regulate rice starch synthesis at HT [11]. Despite these findings, much is still unknown about how the HSP70 and HSP40 families respond to HT stress and participate in regulation of endosperm starch synthesis and grain filling under HT.

Here, we found that the HSPs OsHsp70-2 and OsHsp40-1 form a molecular module that stabilizes two key starch biosynthetic enzymes, granule-bound starch synthase (OsGBSSI) and pyruvate orthophosphate dikinase (OsPPDKB), under moderate HT. Overexpression of *OsHsp70-2* and *OsHsp40-1* increased the activities of GBSSI and PPDKB, elevated starch synthesis, reduced chalky grain rate and increased grain yield significantly in three different genetic backgrounds of rice. Among different rice accessions, natural variations in *OsHsp70-2* and *OsHsp40-1* were associated with differences in rice quality, with the specific alleles present in most *AUS* rice accessions, conferring better rice appearance quality under HT. Our results provide insights into the mechanisms underlying decreased rice quality and yield under HT stress and how rice protects itself against heat stress during the grain-filling stage. It also provides a new theoretical basis and genetic resources for improving rice quality and yield under HT conditions.

## RESULTS

### OsHsp40-1 interacts with OsHsp70-2, enhancing the ATPase activity of OsHsp70-2

**Figure 1.**
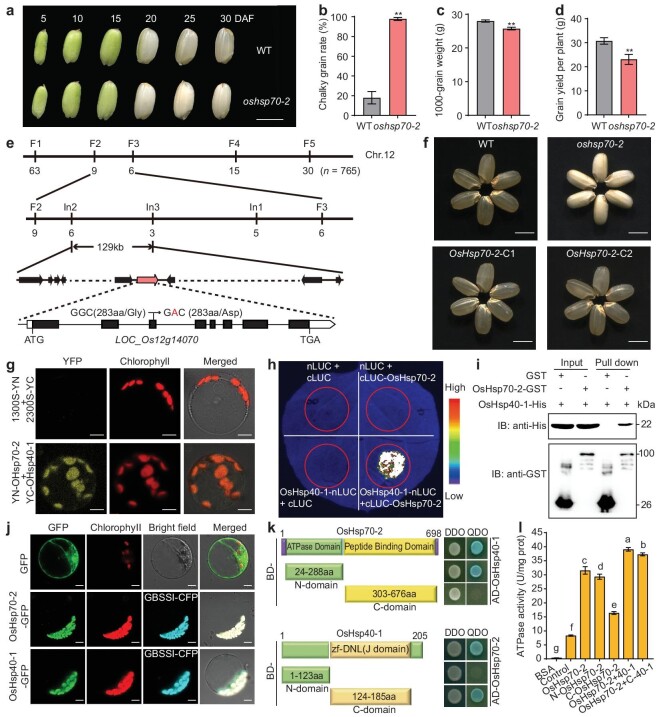
Identification of OsHsp70-2 and its interacting protein OsHsp40-1. (a) Fresh grains of the *oshsp70-2* mutant and wild type (WT) at various stages of development. DAF, days after fertilization. Scale bar, 5 mm. (b–d) Chalky grain rate (b), 1000-grain weight (c) and grain yield per plant (d) of *oshsp70-2* and WT. Data are means ± SD, *n* = 3 (no less than 200 grains per replication) in (b and c), *n* = 10 in (d). Statistical comparisons were performed using Student's *t*‐test (**P < 0.01). (e) Map-based cloning of *OsHsp70-2*. Bottom panel: *OsHsp70-2* (*LOC_Os12g14070*) gene structure and the site mutated in *oshsp70-2*. (f) Grain appearance of WT, *oshsp70-2* and complementation lines (*OsHsp70-2* C1 and C2). Scale bars, 5 mm. All plants were grown in natural HT conditions in Hangzhou, 2020 (daily average temperature 30°C during grain-filling stage). (g) Interaction between OsHsp70-2 and OsHsp40-1 detected by BiFC assays in rice protoplasts. ‘1300S-YN + 2300S-YC’ indicates negative control. Scale bars, 5 µm. (h) Interaction between OsHsp70-2 and OsHsp40-1 detected by LCI assays in *Nicotiana (N.) benthamiana* leaves. (i) Interaction between OsHsp70-2 and OsHsp40-1 detected by glutathione S-transferase (GST) pull-down assays. GST- or GST-OsHsp70-2-coupled magnetic beads were used to pull down Histidine (HIS)-OsHsp40-1 proteins, and proteins detected by anti-HIS or anti-GST antibodies. (j) Co-localization of OsHsp70-2 and OsHsp40-1 with OsGBSSI in rice protoplasts. Scale bars, 50 µm. (k) Determining the core interaction domains of OsHsp70-2 and OsHsp40-1 by yeast two-hybrid (Y2H) assays. Upper panel: interactions between different truncated forms of OsHsp70-2 and full-length OsHsp40-1. Bottom panel: interactions between different truncated forms of OsHsp40-1 and full-length OsHsp70-2. (l) ATPase activities of *E. coli*-expressed full-length and truncated forms of OsHsp70-2 and of the combination of OsHsp70-2 with full-length or truncated forms of OsHsp40-1. BSA, standard protein. Control, GST elution buffer. Data are means ± SD (*n* = 3). Different letters indicate significant differences at *P* < 0.05 by ANOVA and Duncan's test.

To elucidate the molecular mechanisms of endosperm starch synthesis in rice, we identified an opaque endosperm mutant, *oshsp70-2*, from a mutant library of *japonica* cultivar Zhonghua11 (ZH11). When grown in the natural HT environment of Hangzhou in 2020 (daily average temperature 30°C during grain-filling stage, Table S1) we found that *oshsp70-2* showed significantly decreased grain weight and grain yield per plant, with an increased chalky grain rate compared with the wild type (Fig. 1a–d, Fig. S1). We performed map-based cloning of the mutant gene using an F_2_ population generated by crossing *oshsp70-2* with Nanjing11 (*indica*). The *OsHsp70-2* locus was localized within a 129-kb genomic region on Chr. 12, and sequencing revealed a single nucleotide substitution of guanine (G) to adenine (A) in the 3rd exon of *LOC_Os12g14070* in the *oshsp70-2* mutant, resulting in an amino acid conversion from glycine (Gly) to aspartic acid (Asp) at position 283 (Fig. 1e). *LOC_Os12g14070* has been annotated as an HSP [9,10]. To confirm that *LOC_Os12g14070* is responsible for the mutant phenotype, a complementation vector containing the wild-type *LOC_Os12g14070* genomic sequence, including its native promoter, was transformed into *oshsp70-2*. Grains harvested from independent T_1_ complementation lines were restored to a transparent-endosperm phonotype, and there were almost no differences in the other major agronomic traits of the T_1_ plants compared to the wild type (Fig. 1f, Fig. S1). Therefore, *LOC_Os12g14070* (*OsHsp70-2*) is the gene responsible for the *oshsp70-2* phenotype.

To uncover how *OsHsp70-2* regulates rice quality and grain weight, we used yeast two-hybrid (Y2H) assays to find interacting proteins, identifying another HSP, OsHsp40-1 (LOC_Os06g50870), that can interact with OsHsp70-2 (Fig. S2a and b and Table S2). The results of bimolecular fluorescence complementation (BiFC), the firefly luciferase complementation imaging (LCI) system, coimmunoprecipitation (CoIP) assays, and glutathione S-transferase (GST) pull-down analysis all confirmed that OsHsp40-1 can physically interact with OsHsp70-2 both *in vitro* and *in vivo* (Fig. 1g–i; Fig. S2c). OsHsp70-2 includes an N-terminal ATPase domain and a C-terminal substrate binding domain, and OsHsp40-1 has a centrally located DnaJ-Type Zn finger structure (J-domain), suggesting that OsHsp70-2 and OsHsp40-1 are typical Hsp70 and Hsp40 proteins, respectively; and the amino acid substitution in the *oshsp70-2* mutant is located in the ATPase domain of OsHsp70-2 (Fig. S3). Quantitative reverse transcription polymerase chain reaction (RT-qPCR) analysis showed that both *OsHsp70-2* and *OsHsp40-1* were expressed in all examined tissues, with *OsHsp70-2* being abundantly expressed in developing grains, and *OsHsp40-1* expression gradually increased from low to high during grain development (Fig. S4a and b). We also found that the expression levels of *OsHsp70-2* and *OsHsp40-1* significantly increased when seedlings were moved from a normal temperature (28°C) to an HT (42°C) (Fig. S4c and d). Subcellular localization analysis showed that both proteins were located in chloroplasts and co-localized with GBSSI, implying that OsHsp70-2 and OsHsp40-1 are localized in amyloplasts (Fig. 1j).

J-domain proteins are known to interact with the ATPase domain of Hsp70 and stimulate its ATPase activity [12]. Our domain truncation analysis indicated that the N-terminal ATPase domain of OsHsp70-2 and the J-domain of OsHsp40-1 are essential for the interaction between these two HSPs (Fig. 1k). We next examined how OsHsp40-1 affected OsHsp70-2’s ATPase activity *in vitro*, using proteins expressed in *Escherichia coli (E. coli)*. Both full-length OsHsp70-2 and its N-terminal ATPase domain displayed ATPase activity, and the ATPase activity of OsHsp70-2 was significantly increased in the presence of OsHsp40-1 or its J-domain (Fig. 1l; Fig. S4e–g). We further found that the total ATPase enzyme activity in the developing endosperm of *oshsp70-2* was significantly decreased, while that of complementation lines was almost no different compared with the wild type (Fig. S1j). These results imply that OsHsp40-1 and OsHsp70-2 may function together as co-chaperones to influence rice growth and development.

### Mutation of *OsHsp70-2* and *OsHsp40-1* compromises rice quality and grain yield, especially at high temperatures

**Figure 2.**
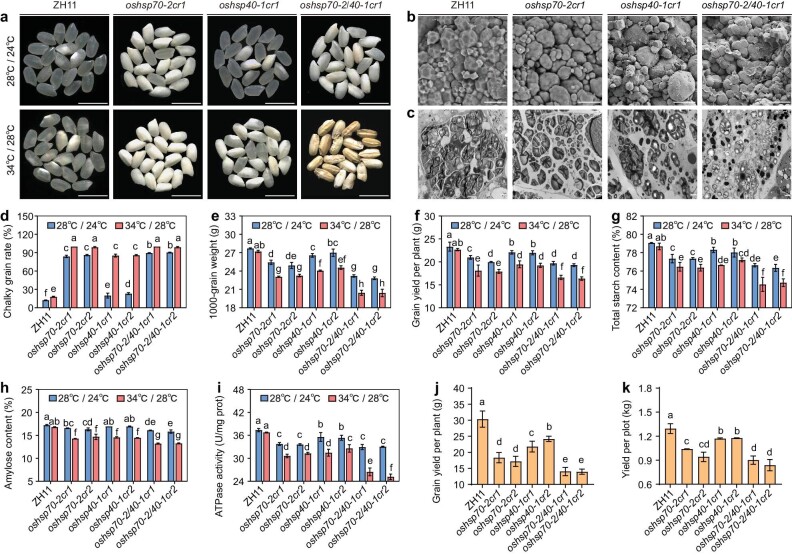
Mutation of OsHsp70-2 and OsHsp40-1 compromises rice quality and grain yield, especially at HT. (a) Appearance of milled rice of WT (ZH11), single and double mutants of *OsHsp70-2* and *OsHsp40-1* plants grown under normal-temperature conditions (NT; 28°C, 12 h light/24°C, 12 h dark) and artificial HT conditions (HT; 34°C, 12 h light/28°C, 12 h dark) during the grain-filling stage. Scale bars, 10 mm. (b) Scanning electron microscopy of the central area of mature grain endosperm of WT and mutants under HT conditions. Scale bars, 10 μm. (c) Transmission electron microscopy images of transverse sections of 9 DAF grains of WT and mutants under HT conditions. Scale bars, 2 μm. (d–h) Chalky grain rate (d), 1000-grain weight (e), grain yield per plant (f), endosperm total starch content (g) and amylose content (h) of WT and mutants under NT and HT conditions. (i) The ATPase activity in 10 DAF endosperm of WT and mutants under NT and HT conditions. (j and k) The grain yield per plant and yield per plot of WT and mutants under natural HT conditions in Hangzhou, 2021 (daily average temperature 28.4°C during the grain-filling stage). Data in (d–k) are means ± SD, *n* = 3 in (d, e, g, h, i), *n* = 4 in (f), *n* = 10 in (j), *n* = 2 in (k), and there are no less than 200 grains per replication in (d and e). Different letters indicate significant differences at *P* < 0.05 by ANOVA and Duncan's test.

To further explore the biological functions of *OsHsp70-2* and *OsHsp40-1*, we used CRISPR/Cas9 gene editing to generate the single mutants *oshsp70-2cr* and *oshsp40-1cr*, and the double mutant *oshsp70-2/40-1cr*, in the ZH11 genetic background (Fig. S5). We grew them in the natural HT of Hangzhou in 2021 (daily average temperature 28.4°C during grain-filling stage, Table S1). Compared with the wild type, the mature grains of *oshsp70-2cr* also exhibited opaque endosperm, *oshsp40-1cr* showed chalky endosperm, and the grains of the *oshsp70-2/40-1cr* were floury and shrunken (Fig. S6a–d). Compared with the wild type, the single and double mutants all showed decreased grain weight and grain yield, with significantly reduced starch and protein contents and higher soluble sugar contents in the endosperm (Fig. S6e–l; Fig. 2j and k). Aside from these grain phenotypes, there were no visible differences in plant morphology between the mutants and the wild type (Fig. S6m–r).

As well as being under the natural HT at Hangzhou, during the grain-filling stage, we also moved the plants of wild type, single and double mutants to artificial normal-temperature (NT; 28°C, 12 h light/24°C, 12 h dark) and HT (34°C, 12 h light/28°C, 12 h dark) conditions. We were surprised to find that the endosperm development is more susceptible to HT in the single and double mutants of *OsHsp70-2* and *OsHsp40-1* than in the wild type. In the wild type, the chalkiness rate was significantly increased (12% to 18%) in HT compared to NT conditions, with no significant changes in grain weight, starch content and yield per plant. In contrast, *oshsp70-2cr* grains were floury under HT conditions, and the chalkiness rate rose to almost 100%, with significantly decreased grain weight, starch content and grain yield per plant. Under HT conditions, the chalkiness rate of *oshsp40-1cr1* was almost four times (20% to 85%) that of the plants under NT conditions, and mature grains of the *oshsp70-2/40-1cr1* double mutant became obviously floury and shrunken, with a chalkiness rate of almost 100% and a significantly decreased grain weight, starch content and grain yield per plant (Fig. 2a, d–h; Fig. S7a, e–h).

Under HT, the expression levels of both *OsHsp70-2* and *OsHsp40-1* in wild type and single and double mutants were all significantly higher than those under NT conditions (Fig. S7i and j). The endogenous ATPase activities in *oshsp70-2cr1, oshsp40-1cr1* and *oshsp70-2/40-1cr1* were significantly decreased under HT compared to NT conditions, but were unaffected in the wild type (Fig. 2i). We further compared the morphology of starch grains in the endosperm cells of *oshsp70-2cr, oshsp40-1cr, oshsp70-2/40-1cr* and the wild type under HT conditions. The starch granules (SGs) were polyhedral and tightly packed in the mature endosperm of wild type, but irregularly rounded and loosely packed in the single and double mutants (Fig. 2b; Fig. S7b). In the developing endosperm, the central regions of wild-type endosperm cells were filled with compound starch grains, whereas the endosperm cells of *oshsp70-2cr* and *oshsp40-1cr* had fewer compound grains, and those of the double mutant were filled with almost exclusively single SGs (Fig. 2c; Fig. S7c and d). Taken together, these results indicate that both OsHsp70-2 and OsHsp40-1 play an important role in the regulation of endosperm starch synthesis, thereby affecting grain quality and yield in rice, especially under moderate HT environments.

### Overexpression of *OsHsp70-2* and *OsHsp40-1* improves rice quality and grain yield under natural high-temperature conditions

**Figure 3.**
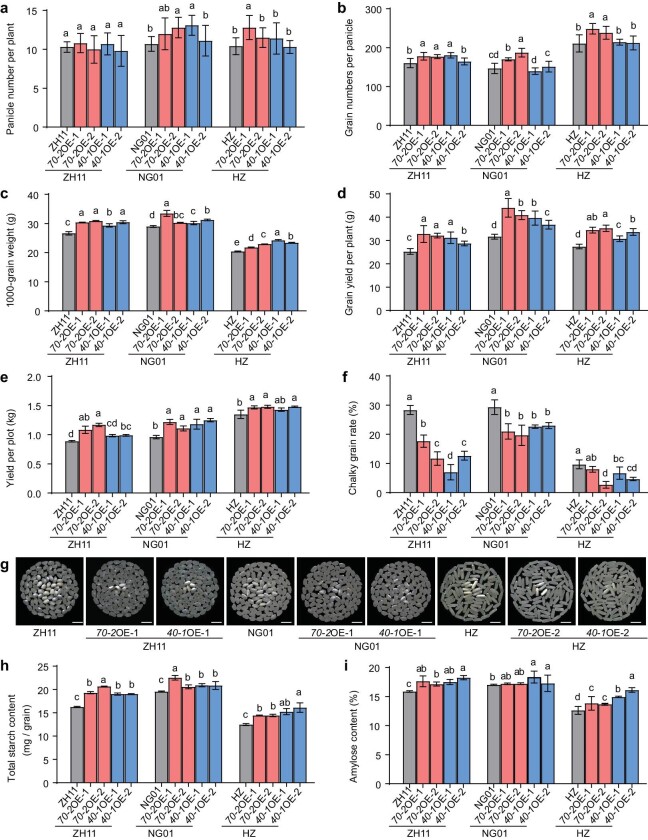
Overexpression of *OsHsp70-2* and *OsHsp40-1* improves rice quality and grain yield. (a–e) Panicle number per plant (a), grain number per panicle (b), 1000-grain weight (c), grain yield per plant (d) and grain yield per plot (e) of OE lines of *OsHsp70-2* and *OsHsp40-1* in the ZH11, NingGeng01 (NG01, *japonica*) and Huazhan (HZ, *indica*) backgrounds. (f) The chalky grain rate of OE lines in the three genetic backgrounds. (g) Appearance of milled rice of OE lines in the three genetic backgrounds. Scale bars, 10 mm. (h and i) Total starch content per grain (h), and % content of amylose (i) in endosperm of OE lines in the three genetic backgrounds. All plants were grown in natural HT conditions in Hangzhou, 2021 (daily average temperature 28.4°C during the grain-filling stage). Data in (a–f, h and i) are means ± SD, *n* = 10 in (a, b, d), *n* = 3 in (c, f, h, i) and *n* = 2 in (e), and there are no less than 200 grains per replication in (c and f). Different letters indicate significant differences at *P* < 0.05 by ANOVA and Duncan's test.

Given that the OsHsp70-2–OsHsp40-1 module is required for maintaining normal endosperm starch synthesis, grain quality and yield, we asked whether overexpressing *OsHsp70-2* and *OsHsp40-1* would improve these traits in elite rice cultivars under moderate HT conditions. We introduced *pUbi : OsHsp70-2^ZH11^* and *pUbi : OsHsp40-1^ZH11^* into wild-type ZH11 as well as Ninggeng01 (NG01, *japonica*) and Huazhan (HZ, *indica*), two elite cultivars that are widely grown in China, to generate *OsHsp70-2* overexpression (OE) (ZH11, NG01 and HZ) and *OsHsp40-1* OE (ZH11, NG01 and HZ) lines. When grown in the naturally HT conditions of Hangzhou in the summers of 2021 and 2023 (Table S1), the *OsHsp70-2* and *OsHsp40-1* OE lines in all three backgrounds had significantly larger and heavier grains, whereas the grain number per panicle and the effective panicle number per plant were unchanged or increased only slightly (Fig. 3a–c; Figs S8–S11). Finally, the grain yield per plant and yield per plot were significantly higher in the *OsHsp70-2* and *OsHsp40-1* OE lines than in the corresponding ZH11, NG01 and HZ wild types (Fig. 3d and e; Fig. S11). Moreover, we found that the *OsHsp70-2* and *OsHsp40-1* OE lines had mature grains with significantly lower chalky grain rate and increased starch contents than those of the corresponding wild type under natural HT in Hangzhou in the summers of 2021 and 2023 (Fig. 3f–i; Fig. S11j). Moreover, we also moved the plants of *OsHsp70-2* and *OsHsp40-1* OE lines to artificial NT (28°C, 12 h light/24°C, 12 h dark) and HT (34°C, 12 h light/28°C, 12 h dark) conditions during the grain-filling stage, and found that the chalky grain rates of *OsHsp70-2* OE and *OsHsp40-1* OE were significantly lower than that of the wild type, with significantly increased total starch contents, grain size and weight, and grain yield per plant under both HT and NT environments (Fig. S12a–j). Taken together, these results demonstrate the great potential of using *OsHsp70*-2 and *OsHsp40*-1 for the genetic improvement of rice quality and yield.

### The OsHsp70-2–OsHsp40-1 module regulates rice quality and grain yield by maintaining the stability of OsGBSSI and OsPPDKB

**Figure 4.**
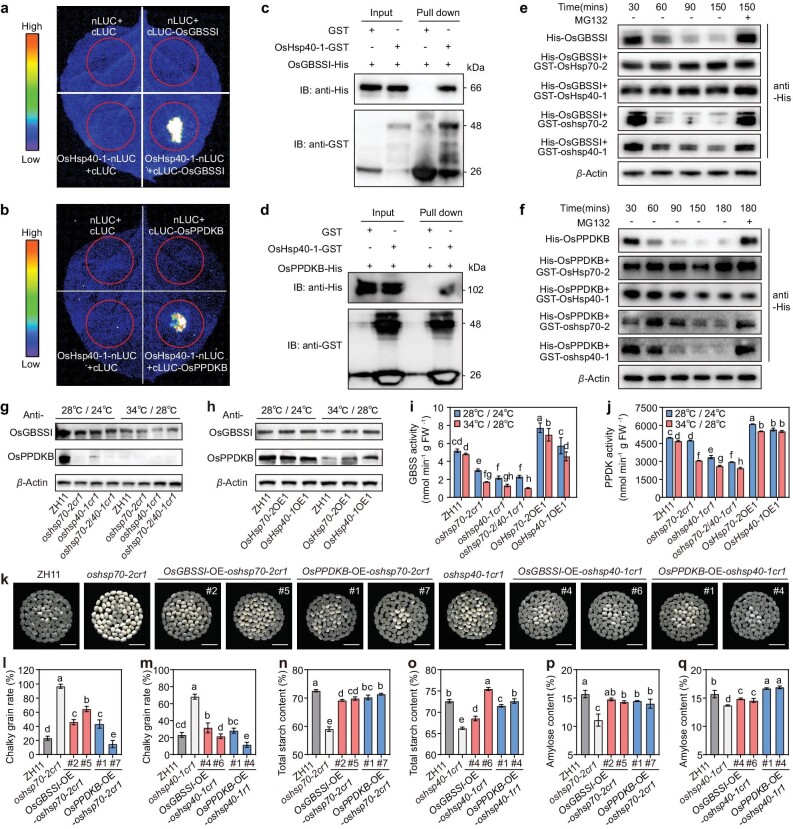
The OsHsp70-2–OsHsp40-1 module functions upstream of OsGBSSI and OsPPDKB to maintain endosperm starch biosynthesis. (a and b) Interaction between OsHsp40-1 and OsGBSSI or OsPPDKB detected by LCI assays in *N. benthamiana* leaves. (c and d) Interaction between OsHsp40-1 and OsGBSSI or OsPPDKB detected by GST pull-down assays. (e and f) Cell-free degradation assay of His-OsGBSSI (e) and His-OsPPDKB (f) in the absence or presence of OsHsp70-2 and OsHsp40-1. The protease inhibitor MG132 was added to suppress degradation of His-OsGBSSI and His-OsPPDKB. Actin was used as an internal control. (g and h) Immunoblot analysis of OsGBSSI and OsPPDKB protein abundance in 10 DAF endosperm of WT, mutants and OE lines of *OsHsp70-2* and *OsHsp40-1* under NT (28°C, 12 h light/24°C, 12 h dark) and HT (34°C, 12 h light/28°C, 12 h dark) conditions. Actin served as a loading control. (i and j) GBSS (i) and PPDK activity (j) in 10 DAF endosperm of WT, mutants and OE lines under NT and HT conditions. (k) The appearance of milled rice of OE lines of *OsGBSSI* or *OsPPDKB* under the *oshsp70-2cr1* or *oshsp40-1cr1* background. Scale bars, 10 mm. (l and m) The chalky grain rate of *OsGBSSI* and *OsPPDKB* OE plants with the *oshsp70-2cr1* (l) or *oshsp40-1cr1* (m) background. (n–q) The content (%) of total starch (n and o) and amylose (p and q) in endosperm of *OsGBSSI* and *OsPPDKB* OE plants. All plants in (k–q) were grown in natural HT conditions in Hangzhou, 2022 (daily average temperature 31.2°C during the grain-filling stage). Data in (i–j, l–q) are means ± SD, *n* = 3, and there are no less than 200 grains per replication in (l, m). Different letters indicate significant differences at *P* < 0.05 by ANOVA and Duncan's test.

To determine the underlying mechanisms by which the OsHsp70-2–OsHsp40-1 module regulates grain quality and yield, we used a Y2H assay to screen for potential OsHsp40-1-interacting proteins. We found that OsHsp40-1 interacted with two important starch biosynthesis enzymes, OsGBSSI and OsPPDKB. However, no interaction was detected between either of these two enzymes and OsHsp70-2 (Fig. S13a and b). LCI, CoIP, BiFC and GST pull-down assays all confirmed that OsHsp40-1 physically interacts with OsGBSSI and OsPPDKB (Fig. 4a–d; Fig. S13c–e). In cell-free protein degradation assays, we found that the degradation of OsGBSSI and OsPPDKB was inhibited in the presence of GST-OsHsp70-2 or GST-OsHsp40-1 (Fig. 4e and f; Fig. S13f and g). We next measured transcript, protein and enzymatic activity levels of OsGBSSI and OsPPDKB in developing endosperm of *oshsp70-2cr, oshsp40-1cr, oshsp70-2/40-1cr* and OE lines under both artificial NT and HT conditions. The expression levels of *OsGBSSI* and *OsPPDKB* in wild type, *oshsp70-2cr1, oshsp40-1cr1* and *oshsp70-2/40-1cr1* were significantly lower under HT compared to NT conditions (Fig. S13h–i). Compared to the wild type, the single and double mutants had significantly decreased protein accumulation and enzymatic activities of GBSS and PPDK at both NT and HT, whereas the protein abundances and enzyme activities of all wild type, mutants and OE lines under HT were significantly decreased compared to those at NT conditions. The HT-induced decreases in protein abundance and enzyme activity were more pronounced in the single and double mutants than in the wild type and *OE* lines (Fig. 4g–j).

Further, we individually overexpressed *OsGBSSI* and *OsPPDKB* in the *oshsp70-2cr* and *oshsp40-1cr* backgrounds. Under natural HT conditions (Hangzhou, 2022), the transcript, protein and enzymatic activity levels of OsGBSSI or OsPPDKB in the overexpression (OE) lines were significantly increased compared to the corresponding mutant background (Fig. S14a–l). Notably, the opaque and chalky endosperm phenotypes of the mutants were largely rescued in the lines overexpressing *OsGBSSI* or *OsPPDKB* (Fig. 4k). Compared with the *oshsp70-2cr* and *oshsp40-1cr* mutants, the OE lines had significantly lower percentages of chalky grains and significantly increased grain weights with higher starch and protein contents in the mature grains (Fig. 4l–q; Fig. S14m–v). These results suggested that the loss of function of *OsHsp70-2* and/or *OsHsp40-1* caused the degradation of the two key starch synthesis enzymes GBSSI and PPDKB, ultimately leading to insufficient endosperm starch synthesis, poor rice quality and lower grain yield, and these damages were worse at HT. Together, our findings demonstrated that the OsHsp70-2–OsHsp40-1 module and its downstream effects on OsGBSSI and OsPPDKB are crucial for endosperm starch synthesis and help improve rice quality and grain yield, especially in HT environments.

### Natural variations in *OsHsp70-2* and *OsHsp40-1* contribute to grain appearance quality in rice under natural high-temperature conditions

**Figure 5.**
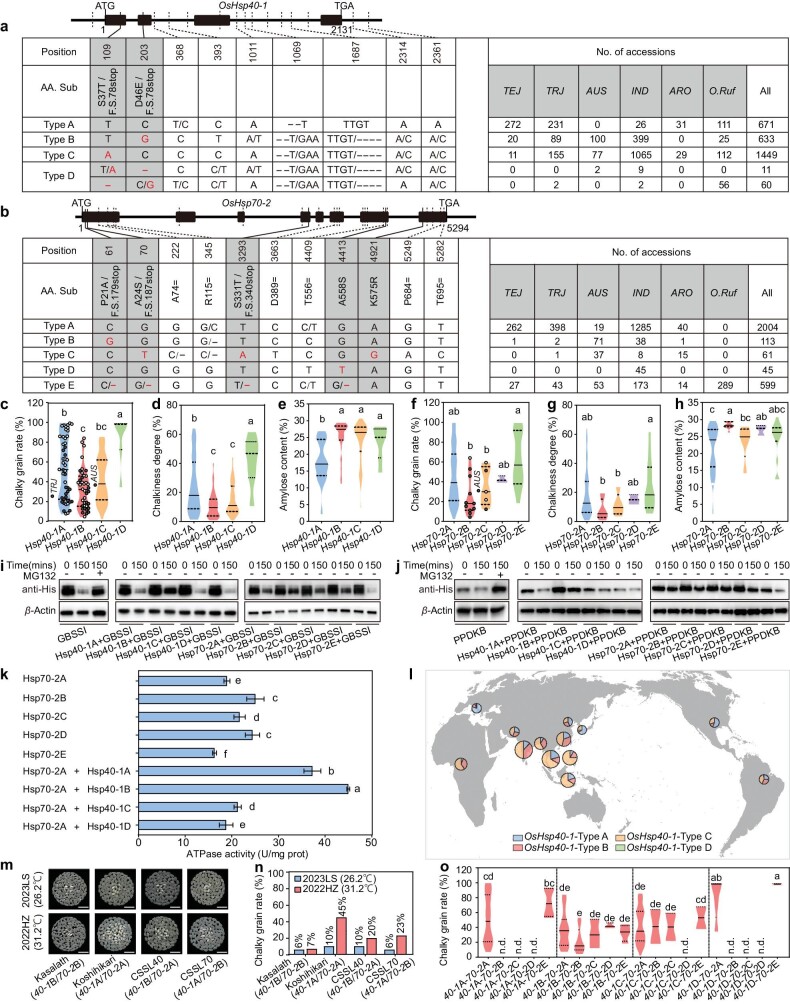
Natural variation of *OsHsp70-2* and *OsHsp40-1*. (a and b) Haplotype analysis of *OsHsp40-1* (a) and *OsHsp70-2* (b) among ∼3000 rice landraces and 300 *O. rufipogon* accessions based on public resequencing data. *OsHsp40-1* and *OsHsp70-2* alleles were grouped into four and five types, respectively. Rice subgroups: *TEJ*, temperate *japonica; TRJ*, tropical *japonica; AUS; IND, indica*; and *ARO*, aromatic population. (c–h) Chalky grain rate (c and f), chalkiness degree (d and g) and amylose content (e and h) of 155 rice landraces with different *OsHsp40-1* and *OsHsp70-2* haplotypes under field HT conditions in Hangzhou, 2022 (2022HZ, daily average temperature 31.2°C during the grain-filling stage). The *TRJ* accessions with the *Hsp40-1*A haplotype and *AUS* accessions with the *Hsp40-1*B haplotype are indicated by solid dots in (c). (i and j) Cell-free degradation assay of His-OsGBSSI (i) and His-OsPPDKB (j) in the absence or presence of GST-OsHsp40-1 (type A, B, C or D) or GST-OsHsp70-2 (type A, B, C, D or E). (k) ATPase activities of the five different GST-OsHsp70-2 types and of OsHsp70-2A combined with the four different GST-OsHsp40-1 types. (l) Geographic distributions of ∼3000 rice landraces carrying different types of *OsHsp40-1* alleles. (m and n) The appearance of milled rice (m) and chalky grain rate (n) of ‘Kasalath’ (an *AUS* rice with *OsHsp40-1*B and *OsHsp70-2*B), ‘Koshihikari’ (a *TEJ* accession with *OsHsp40-1*A and *OsHsp70-2*A) and two chromosome segment substitution lines (CSSL40 and CSSL70, Koshihikari background containing chromosome segments from Kasalath with *OsHsp40-1*B and *OsHsp70-2*B, respectively) under field NT conditions (Lingshui, Hainan province in spring 2023, daily average temperature 26.2°C during the grain-filling stage, 2023LS) and field HT conditions (2022HZ). Scale bars, 10 mm in (m). (o) Chalky grain rate of rice landraces with various combinations of *OsHsp40-1* and *OsHsp70-2* haplotypes in 2022HZ. In (c–h, k, o), different letters indicate significant differences at *P* < 0.05 by ANOVA and Duncan's test. Review drawing number: GS 京 (2025) 0018号

We investigated the natural variation of *OsHsp70-2* and *OsHsp40-1* among ∼3000 rice landraces and ∼300 wild rice (*Oryza rufipogon*) accessions based on public resequencing data [13,14]. According to the nucleotide and amino acid sequence polymorphisms in these data, *OsHsp40-1* has ∼13 haplotypes, which can be grouped into 4 types (*OsHsp40-1*A, B, C and D), and *OsHsp70-2* has ∼14 haplotypes, which can be grouped into 5 types (*OsHsp70-2*A, B, C, D and E). *OsHsp40-1*D and *OsHsp70-2*E, which comprise non-functional alleles with frameshift mutations and premature stops, and the above wild-type ZH11, fall into type of *OsHsp40-1*A and type of *OsHsp70-2*A (Fig. 5a and b; Fig. S15a and b). To determine the association between the *OsHsp40-1* or *OsHsp70-2* genotypes and rice quality traits, we collected 155 rice varieties from a wide geographic range worldwide and grew them in the natural HT environment of Hangzhou in 2022 (daily average temperature 31.2°C during grain-filling stage; Table S1). We found that accessions with *OsHsp40-1*B (mainly carried by *AUS* and *indica* accessions) or *OsHsp40-1*C (mainly carried by *indica* rice and *O. rufipogon*) alleles had significantly lower chalky grain rates and chalkiness degree, with higher grain amylose contents (Fig. 5c–e; Table S3). For *OsHsp70-2*, the chalky grain rates and chalkiness degree were relatively high in the accessions with *Hsp70-2*A (carried by most rice landraces) or *Hsp70-2*E alleles (carried by most *O. rufipogon* and some indica rice) and relatively low in the accessions with *Hsp70-2*B, C (mainly carried by *AUS* rice) and D (carried by some *indica* rice) alleles (Fig. 5f–h; Table S3). However, there were no significant differences in grain weight and protein content among accessions with different alleles of *OsHsp40-1* and *OsHsp70-2* (Fig. S16a–d).

In addition, we tested the ability of the protein variants of different alleles of *OsHsp40-1* and *OsHsp70-2* to stabilize OsGBSSI and OsPPDKB in *in vitro* protein degradation assays. The result showed that the degradation rate of both OsGBSSI and OsPPDKB was slowest in the presence of GST-OsHsp40-1B or GST-OsHsp70-2B, C and D, and not inhibited by GST-OsHsp40-1D or GST-OsHsp70-2E, which are loss-of-function variants (Fig. 5i and j). We also examined the ATPase activities of the OsHsp70-2 protein variants, and found that compared to OsHsp70-2A, OsHsp70-2B, C and D had higher ATPase activity, and the non-functional OsHsp70-2E had significantly lower activity. Moreover, the addition of OsHsp40-1A, B and C significantly increased the ATPase activity of OsHsp70-2A (Fig. 5k).

Interestingly, we noticed that most *AUS* rice accessions have *OsHsp40-1*B or C alleles and *OsHsp70-2*B or C alleles and have lower chalky grain rates and chalkiness degree when grown in the natural HT environment of Hangzhou in 2022 (Fig. 5a–h). This result is consistent with the fact that *AUS* type rice mainly originated at low latitudes and has relatively high heat tolerance. We also analyzed the geographic distributions of ∼3000 rice landraces carrying different types of *OsHsp40-1* and *OsHsp70-2* alleles. The proportion of *OsHsp40-1*A haplotypes was relatively high in rice landraces from high latitudes, whereas the proportion of *OsHsp40-1*B and C haplotypes was relatively high in landraces from low latitudes (Fig. 5l). The proportion of *OsHsp70-2*B and C haplotypes was relatively high in landraces from India, Bangladesh and Pakistan (Fig. S15c). It suggested that there was a regional differentiation in haplotypes of *OsHsp40-1* and *OsHsp70-2* and the *OsHsp40-1*B or C and *OsHsp70-2*B or C carried by most landraces in low latitudes possibly contribute to their higher tolerance to HT during the grain-filling stage.

We noticed that an elite temperate *japonica* cultivar ‘Koshihikari’ with *OsHsp40-1*A and *OsHsp70-2*A haplotypes had a low (10%) chalky grain rate under the NT conditions, but a dramatically higher (45%) chalky grain rate under the HT conditions. In contrast, the *AUS* rice ‘Kasalath’, which originated in the low latitudes of India and has *OsHsp40-1*B and *OsHsp70-2*B haplotypes, had a relatively low chalky grain rate under both natural HT conditions at Hangzhou and natural NT conditions at Hainan Lingshui (26.2°C during the grain-filling stage in the spring of 2023; Table S1) (Fig. 5m and n). Remarkably, when the chromosome segment containing *OsHsp40-1*A or *OsHsp70-2*A in Koshihikari was replaced with the corresponding Kasalath chromosome segment and grew the resulting chromosome segment substitution lines (CSSLs) under natural HT conditions at Hangzhou, the chalky grain rates of CSSL40 (with *OsHsp40-1*B) and CSSL70 (with *OsHsp70-2*B) were significantly lower (20% and 23%, respectively) than that of the Koshihikari (Fig. 5m and n; Fig. S16e–o). We also found that under the HT conditions at Hangzhou, many landraces containing *OsHsp40-1*B or C and *OsHsp70-2*B, C or D alleles together had good grain appearance quality with lower chalky grain rates, and many landraces containing *OsHsp40-1*A or D and *OsHsp70-2*A or E alleles together had higher chalky grain rates (Fig. 5o; Fig. S17). Taken together, these results suggested that natural variations in *OsHsp70-2* and *OsHsp40-1* contribute to variation in grain appearance quality in rice, especially under HT environments, and the favorable alleles of *OsHsp70-2* and *OsHsp40-1* in *AUS* accessions could be used for improving rice thermotolerance during the grain-filling stage.

## DISCUSSION

**Figure 6.**
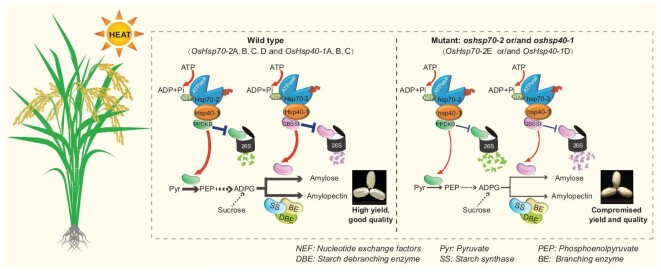
Proposed working model of the OsHsp70-2–OsHsp40-1 module. In wild type (rice varieties with functional haplotype *OsHsp70-2*A, B, C, D and *OsHsp40-1*A, B, C), OsHsp40-1 interacts with OsHsp70-2 and stimulates its ATPase activity, forming a molecular module that targets the key starch synthesis enzymes OsGBSSI and OsPPDKB and enhances their stabilities and enzyme activities during grain filling, thereby conferring better rice quality and grain yield under HT conditions. However, mutation of *OsHsp70-2* and/or *OsHsp40-1* (or rice varieties carrying non-functional haplotype *OsHsp70-2*E and *OsHsp40-1*D) compromises their ATPase activity and the stabilities and enzyme activities of OsGBSSI and OsPPDKB, leading to insufficient endosperm starch biosynthesis and poor grain quality and yield under HT conditions.

Starch is a carbohydrate that accounts for >85% of the dry weight in rice grains, and its biosynthesis is directly related to rice quality and yield. Sucrose produced in leaves is imported into developing grains and used as a carbon source for starch synthesis in the amyloplasts of endosperm cells. Starch biosynthesis is a complex process involving a series of enzymes [15]. GBSSI is the most important enzyme responsible for amylose synthesis and thus directly affects grain quality and yield in rice [16,17]. OsPPDKB is an important modulator of carbon flow for starch biosynthesis during rice grain filling [18]. Extensive research has shown that many important genes controlling starch synthesis and grain quality in rice mainly function by targeting GBSSI and/or OsPPDKB [19,20]. In this study, we found that the OsHsp70-2–OsHsp40-1 module plays a critical role in starch biosynthesis during grain filling, ultimately affecting rice quality and yield, by modulating GBSSI and OsPPDKB (Figs 4, 6). Previous studies in yeast and animals have reported that the J-domain protein Hsp40 promotes Hsp70’s chaperone function by stimulating its ATPase activity [21,22], but the related mechanisms have rarely been studied in plants. Although OsHsp70-2 has been reported to affect rice quality [9,10], the functions of OsHsp40-1 and the molecular mechanisms by which OsHsp70-2 and OsHsp40-1 might regulate rice quality and yield have not been reported. Here, we demonstrate that OsHsp40-1 interacts with OsHsp70-2 and stimulates its ATPase activity (Fig. 1), forming a molecular module that targets the key starch synthesis enzymes OsGBSSI and OsPPDKB and enhances their stabilities and enzyme activities during grain filling, thereby ultimately controlling rice quality and yield (Figs 4, 6). These results enhance our understanding of the genetic basis for HSP-mediated regulation of endosperm starch synthesis, grain quality and yield.

Excessive temperatures and heat stress strongly affect the growth and development of rice. HT at the grain-filling stage usually leads to insufficient accumulation of grain storage compounds and serious chalkiness, in turn causing poor quality and lower yield. And previous studies have shown that this may be due to the disturbance of the expression of genes related to starch synthesis and metabolism, the decrease in activities of starch synthase enzymes and the increase in activities of starch degrading enzymes in HT conditions [23–26]. However, the precise molecular mechanisms underlying rice responses to HT during the filling stage and the HT-induced deterioration of rice quality need to be further explored. Previous studies have shown that Hsp70 and Hsp40 are associated with stresses such as drought, salt and heat during the seedling stage [27,28], with few exploring how Hsp70 and Hsp40 affect rice quality and grain yield under heat stress. Here, we found that the mutants of OsHsp70-2 and OsHsp40-1 were more sensitive than their wild type to HT stress (Fig. 2), while the rice quality and grain-yield phenotypes of *OsHsp70-2* and *OsHsp40-1* OE lines in different genetic background were better than those of the wild type under natural HT conditions (Fig. 3; Figs S8–S11). We further found that the protein levels and enzyme activities of OsGBSSI and OsPPDKB in endosperm were significantly reduced under HT conditions, ultimately compromising starch biosynthesis, and decreasing grain weight (Fig. 4). The presence of functional OsHsp70-2 and OsHsp40-1 in wild-type and OE plants increased the endogenous ATPase activity and enhanced the stability and enzyme activities of OsGBSSI and OsPPDKB under HT conditions, which promoted endosperm starch biosynthesis and ultimately improved heat tolerance during the grain-filling stage (Figs 4h–j, 6; Fig. S8j–l). Therefore, our results reveal a novel mechanism by which the OsHsp70-2–OsHsp40-1 module ameliorates rice heat tolerance during the grain-filling stage.

The heat tolerance of rice usually varies with origin region or variety type. For example, exposure to HT (45°C for 72 h) causes near-wilting of seedlings of *japonica* cultivar Nipponbare, while seedlings of *indica* variety HT54 can tolerate temperatures up to 48°C for 79 h [29,30]. HT (38°C) reduced the number of pollen grains on the stigma in the *AUS* type N22 by 55% and the *japonica* type Moroberekan by 86%, but not the *indica* type IR64 [31]. The *indica* rice 93-11 is more heat tolerant than the temperate *japonica* rice Dongjing during grain filling, which may be related to the alternative splicing of transcription factor OsbZIP58 [32]. However, the more detailed genetic mechanism that led to differences in heat tolerance among rice varieties, especially during grain filling, remains largely unknown. Here, we found that natural variations in *OsHsp70-2* and *OsHsp40-1* are associated with variations in grain chalkiness of rice accessions under HT conditions. Landraces containing *OsHsp40-1*B or C and *OsHsp70-2*B or C haplotypes, such as most *AUS*-type rice, had better thermotolerance and grain appearance quality with lower chalky grain rates under natural HT conditions (Fig. 5a–h). We also found there is a regional differentiation in *OsHsp40-1* and *OsHsp70-2* haplotypes; the proportion of *OsHsp40-1*A haplotype was higher in landraces from high latitudes, whereas the proportion of *OsHsp40-1*B and C was higher in landraces from low latitudes (Fig. 5l). This suggests that varieties from low latitudes have better rice quality than those from high latitudes under HT stress during the grain-filling period, perhaps because of their different *OsHsp40-1* genotypes. A higher proportion of *OsHsp70-2*B and C haplotypes was found in landraces from India, Bangladesh and Pakistan (Fig. S15c), which may be related to the frequent occurrence of HT stress in these areas during the grain-filling stage of rice [33]. We also confirmed that the *OsHsp40-1*B and C and *OsHsp70-2*B and C haplotypes of *AUS-*type rice could serve as genetic resources for breeding HT-tolerant rice varieties (Fig. 5m and n).

In summary, our findings reveal a new mechanism for heat tolerance in rice: during heat stress, the OsHsp70-2–OsHsp40-1 module acts to maintain rice grain starch biosynthesis, thereby improving rice quality and grain yield under HT conditions. Furthermore, overexpression of OsHsp70-2 and OsHsp40-1 and favorable alleles of these two genes provide potential for the genetic improvement of rice quality and yield under HT conditions.

## METHODS

The growth conditions of all rice materials, physicochemical property determination of rice grains, microscopy, gene-map-based cloning, vector construction, rice transformation, RNA extraction, real-time RT-PCR analysis, protein extraction, western blotting, subcellular localization analysis, ATPase, GBSS, PPDK enzyme activity assays, Y2H, BiFC, LCI, pull-down and Co-IP assay, cell-free protein degradation assay, and gene haplotype analysis are described in detail in the supplementary data.

## Supplementary Material

nwae416_Supplemental_File
