# Supplementary material for: A molecular module improves rice grain quality and yield at high temperatures
Source: Natl Sci Rev. 2024 Nov 26;12(2):nwae416. doi: 10.1093/nsr/nwae416 (PMC11759936; doi:10.1093/nsr/nwae416)
Supplement: nwae416_Supplemental_File [file nwae416_supplemental_file.pdf]

Supplemental Materials for

A molecular module improves rice grain quality and yield at high temperature

Feifei Lu[1], Guiai Jiao[1], Jiehua Qiu[1], Shaolu zhao[1], Fengli Zhao[1], Ping Wang[1], Luna Chen[1], Pengfei Chen[1], Xinwei Li[1], Nannan Dong[1], Ruijie Cao[1], Xiaoxue Li[1], Zheyan Ruan[1], Gaoneng Shao[1], Shikai Hu[1], Zhonghua Sheng[1], Lihong Xie[1], Shaoqing Tang[1], Peisong Hu[1,*], and Xiangjin Wei[1,*]

[1] State Key Laboratory of Rice Biology and Breeding, China National Center for Rice Improvement, China National Rice Research Institute, Hangzhou 310006, China

* Correspondence: Xiangjin Wei (weixiangjin@caas.cn), Peisong Hu (hupeisong@caas.cn)

This PDF file includes:

Methods

References for Supplementary Materials

Supplementary Fig. 1 to 17

Supplementary Tables 1 to 4

Methods:

Plant material and growth conditions

The elite cultivars Zhonghua11 (ZH11, *japonica*), Nanjing11 (NJ11, *indica*), Ninggeng01 (NG01, *japonica*), Huazhan (HZ, *indica*) are obtained from China National Rice Research Institute. An opaque endosperm mutant *oshsp70-2* was obtained from a mutant library induced by ethyl methanesulfonate (EMS) treatment of *japonica* ZH11. The CRISPR/Cas9 mutants and overexpression (OE) lines were obtained by this study. 155 rice varieties from a wide geographic range of the world were collected by our laboratory. All these materials were grown in the experimental field in Hangzhou, Zhejiang province or in Lingshui, Hainan province, China. The average daily temperature during grain filling stage of rice was shown in the Supplementary Table 1. Sowing for ZH11, mutants, and complementation lines in the experimental field in Hangzhou was carried out about 25 May, while sowing for 155 rice varieties and OE lines of *OsHsp70-2*, *OsHsp40-1* in HZ, NG01 and ZH11 was carried out about 15 May. At least 20 seedlings of each entry, and two plots with 120 seedlings of each plot for the plot yield test were transplanted into the field with an interplant separation of 17 cm and an inter-row separation of 27 cm. Field management, including irrigation, fertilization and pest control followed commercial practices. At the flowering stage, some well-grown ZH11, mutants, and OE plants were moved to plant growth chambers under normal-temperature (28℃, 12 h light/24℃, 12 h dark; light intensity, 25000LX; humidity, 75%) and high-temperature (34℃, 12 h light/28℃, 12 h dark; light intensity, 25000LX; humidity, 75%) conditions.

Investigation of agronomic traits

The main agronomic traits including plant height, tiller number, grain number per panicle, seed setting rate, grain shape, 1,000-grain weight, grain yield per plant, grain yield per plot, grain chalky rate, and chalkiness degree etc., were analyzed used the mature grains. For agronomic traits such as plant height, tiller number, and yield per plant, statistics were conducted on at least 10 independent plants (n=10) in field experiment, and 4 or 6 independent plants (n=4 or 6) in plant growth chambers under normal-temperature and high-temperature conditions. For complex traits like thousand grain weight, chalkiness rate, and chalkiness degree, three independent biological replicates were performed (n=3), with each replicate consisting of at least 200 grains. For the plot yield, the marginal plants were removed, and only the most central 50 plants of each plot were collected, and the total grain yield of the 50 plants was measured as the plot yield (n=2). Other agronomic traits were analyzed by conventional methods.

Physicochemical properties of rice grains

The total starch percentage content of grain endosperm was measured by a starch assay kit (Megazyme, Wicklow, Ireland) according to the manufacturer's protocol. The total starch in per grain (mg/grain) were also measured for each genetic material. First, we measured the starch and amylose content (mg) in 0.05 g of rice flour, and converted 0.05 g of rice flour into the number of grains according to the 1000-grain

weight, and finally obtain the total starch content in each grain (mg/grain). Amylose percentage content was measured using the spectrophotometric method (NY/T 2639–2014, the Ministry of Agriculture of the People's Republic of China, 2014), and the Infinite M200 PRO plate reader (Tecan, Männedorf, Switzerland) was used to replace the spectrophotometer. Protein contents were measured following the reported by Kang et al.[1].

Microscopy

Scanning electron microscopy (SEM) was performed as described previously[2]. Samples were observed with a HITACHI S–3400N scanning electron microscope. (Hitachi, Tokyo, Japan). For observation of the development of compound starch granules, transverse sections of endosperms at 10 DAF were used to prepare samples of semi-thin sections. Samples were stained with toluidine blue for 5 seconds, then rapid observation under a light microscope (Nikon Eclipse 80i), followed by observation as described previously[2]. For analyzing the ultrastructure of amyloplasts, transverse sections of developing grain (10 DAF) were fixed overnight in 100 mM phosphate buffer (pH 7.2) with 2.5% (v/v) glutaraldehyde and 1% (w/v) paraformaldehyde. Samples were treated as described by Li et al.[3] and observed by transmission electron microscope (TEM) (Hitachi H-7650).

Map-based cloning

To map the *OsHsp70-2* locus, we constructed an F_2 population derived from a cross between the *oshsp70-2* mutant and NJ11, and using approximately 180 polymorphic SSR markers equally dispersed over the whole genome. Further molecular markers were developed based on the nucleotide polymorphisms in the corresponding regions between the ZH11 and NJ11. A total 856 F_2 individuals with floury endosperm seeds phenotype were used for mapping of the *OsHsp70-2* gene. All materials were grown under natural high-temperature conditions in the Hangzhou,2020, the daily average temperature during the grain filling period was 30°C, as shown in Supplementary Table 1. Primers used for mapping are listed in Supplementary Table 4.

Vector construction and rice transformation

The wild-type full length genomic fragment (including the downstream 114bp region) of the *OsHsp70-2* under control of its native promoter (the upstream 1886bp segment from the start codon) from ZH11 was amplified by PCR then cloned into the binary vector pCAMBIA1300 to generate the complementation vector. The wild-type *OsHsp70-2* cDNA or *OsHsp40-1* cDNA sequence driven by *UBIQUTIN1* promoter was cloned into the binary vector pCAMBIA1390 to generate overexpression vector. CRISPR/Cas9 system was used for targeted genome modification of crops to create knockout mutants[4], single guide RNAs targeting sites were constructed into the BGK03 vector (Biogle, Hangzhou, China). All the primers used in plasmid constructs are listed in Supplementary Table 4. For rice transformation, various expression constructs were generated and introduced into the calli generated from mature seed embryos of ZH11 or mutants by *Agrobacterium*-mediated transformation.

RNA extraction and real-time RT-PCR analysis

Total RNA was extracted from different tissues (root, stem, leaf, panicle and developing grains at 5, 10,15,20 and 25 DAF) using the Trizol reagent (Life technologies, Carlsbad, CA, USA). The first-strand cDNA was synthesized from 2 μg of total RNA was reverse transcribed by priming with oligo-dT (20) primer based on the ReverTra Ace qPCR RT Kit (Toyobo, Osaka, Japan). Quantitative PCR (qRT–PCR) was performed using the SYBR Green Real-time PCR Master Mix (Toyobo). The transcription levels were calculated by the $2^{-\Delta\Delta CT}$ values with the expression of *Actin1* gene (accession number AK100267) as the internal control. The primer sequences used in this analysis are listed in Supplementary Table 4.

Protein extraction and western blotting

Protein extraction was conducted in extraction buffer consisting of 25 mM Tris-HCl, pH 7.4, 150 mM NaCl,1 mM EDTA, 1% NonidetP-40, 5% glycerol, 1 mM PMSF, 1X protease inhibitor cocktail. Total protein in the endosperm 10-DAF and 14-days seedling were extracted as described above and separated by SDS-PAGE gels. The proteins were transferred to polyvinylidene fluoride (PVDF) membrane that active by methanol. The target protein bands were detected with the corresponding antibodies. The dilutions of the primary antibodies against GBSSI, PPDKB and ACTIN (CWBIO, Beijing, China) were 1:2000, 1:4000 and 1:5000, respectively, at 4℃ overnight, and secondary antibody with HRP (CWBIO) for 1 h. The immunoblot signal was detected using SuperSignal West Pico Chemiluminescent Substrate (Thermo, Waltham, USA) and visualized by the ChemiDocTM MP Imaging System (Bio-Rad, CA, USA).

Subcellular localization analysis

The full-lengths of *OsHsp70-2*, *OsHsp40-1*, *OsPPDKB* and *OsGBSSI* without a stop codon were cloned into the pAN580-GFP or pCAMBIA1305-GFP. The fusion constructs and control vector were expressed in rice protoplasts and incubated in dark at 28°C for 2 days following previous description[5].The full-lengths of *OsHsp40-1*, *OsPPDKB* and *OsGBSSI* without a stop codon were cloned into the pA7-CFP for co-localization analysis. Rice protoplasts were co-transfected with *35S::OsGBSSI:eGFP/35S::OsHsp40-1:CFP*, *35S::OsHsp70-2:eGFP/35S::OsGBSSI:CFP*, *35S::OsHsp70-2:eGFP/35S::OsGBSSI:CFP*, and *35S::OsPPDKB:eGFP/35S::OsGBSSI:CFP* (at a ratio of 1:1), incubated in W5 solution for 12 h. GFP fluorescence signals were detected using a Zeiss LSM710 confocal laser scanning microscope (Zeiss, Oberkochen, Germany). Excitation laser wavelengths of 439 nm and 488 nm were used for imaging CFP and GFP signals, respectively. Images presented in the figures are representative of at least three protoplasts. The primer sequences used in this analysis are listed in Supplementary Table 4.

ATPase, GBSS, and PPDK enzyme activities assays

The CDS of *OsHsp70-2*, *N-OsHsp70-2*, *C-OsHsp70-2*, *OsHsp40-1* and *C-OsHsp40-1* were amplified and cloned into vector pGEX-4T-1. The resulting constructs were transformed into Escherichia coli strain Rossetta DE3 (BL21). Recombinant protein expression was induced by adding 0.5 mM isopropylthio-β-D-galactoside and and purified by the GST-Sefinose Kit (BBI Life science, Shanghai, China) according to

the manufacturer's protocols. Both fresh leaf tissues from 14-day seedings of wild-type, knockout mutants, overexpression transgenic lines and recombinant protein (GST-tag) were used to measure ATPase activity by the ATPase/GTPase Activity Assay Kit (Sigma-Aldrich, Taufkirchen, Germany) according to the manufacture's instruction. Endosperm samples at 10 DAF were derived and homogenized on ice in extraction buffer (25 mM Tris-HCl, pH7.4, 150 mM NaCl, 1 mM EDTA, 1% NonidetP-40, 5% glycerol, 1 mM PMSF, 1× protease inhibitor cocktail). The resulting supernatant was used for further enzyme activity analysis. The activity of PPDK was measured using a PPDK Assay Kit (Keming, Suzhou, China). GBSSI activity was based on a GBSS Assay Kit (Solarbio, Beijing, China).

Y2H, BiFC, and LCI assay

For Y2H, the full-length coding sequences or its truncations of *OsHsp70-2*, *OsHsp40-1*, *OsPPDKB*, and *OsGBSSI* were cloned into prey vector pGADT7 or the bait vector PGBKT7 (Clontech, Dalian, China). Yeast transformation was performed according to the manufacturer's instructions of Matchmaker GAL4 Two-Hybrid System (Clontech). For BiFC, the full-length coding sequence of *OsHsp40-1*, *OsHsp70-2*, *OsPPDKB* and *OsGBSSI* were amplified by PCR and cloned into the binary vectors pCAMBIA2300S-YC or pCAMBIA1300S-YN to form the cYFP-protein and nYFP-protein constructs, respectively. Subsequently, different combinations constructs were transformed into rice protoplasts according to the protocols described by previous study[6]. YFP fluorescent signals were detected using a ZeissLSM710 confocal laser scanning microscope (CarlZeiss AG, Jena, Germany). For Luciferase complementation imaging (LCI) assay, the CDS of *OsHsp40-1* was cloned into pCAMBIA1300-nLUC, while the *OsHsp70-2*, *OsPPDKB* and *OsGBSSI* were cloned into pCAMBIA1300-cLUC, yielding the OsHsp40-1-nLUC and cLUC-OsHsp70-2/OsPPDKB /OsGBSSI constructs for the LCI assay, following a previously described protocol[7]. Primers used in Y2H, BiFC, and LCI assay are listed in Supplementary Table 4.

Pull-down and Co-IP assay

The cDNA sequences of *OsHsp40-1* was cloned into the pET28a or pGEX-4T-1 vectors, respectively. The *OsHsp70-2* cDNA was inserted into the pGEX-4T-1 vector. The cDNA sequence of *OsPPDKB* and *OsGBSSI* were all inserted into the pET28a. The empty and recombinant plasmids were transformed into *Escherichia coli* DE3 cells to express the GST or His fused protein. Briefly, the *E.coli* cells harboring GST fusion protein were induced by 0.1 mM isopropyl β-D-1-thiogalactopyranoside (IPTG) at 24°C for 16 h and then extracted and purified using the GST-Sefinose Kit (BBI Life science, Shanghai, China). The soluble His fusion protein was induced with 0.5 mM IPTG at 20°C for 16 h and purified using His-tagged protein purification kit (CWBIO, Beijing, China). GST or OsHsp70-2-GST coupled beads were used to capture His or OsHsp40-1-His and then their interaction was detected by anti-His antibodies. GST or OsHsp40-1-GST coupled beads were used to capture OsPPDKB-His, OsGBSSI-His, respectively. Protein-protein interaction was detected by anti-His antibody (Beyotime, Shanghai, China).

For Co-IP assay, the full-length cDNAs of *OsHsp70-2*, *OsHsp40-1*, *OsPPDKB*, and *OsGBSSI* were cloned into the binary vectors pYBA1132-EGFP and/or pHY35S (3×Flag), respectively. Transformation of all constructs into *Agrobacterium* strain EHA105. The positive *Agrobacterium* clones containing the correct constructed plasmids were cultured and co-infiltrated into *N. benthamiana* leaves. Total protein was extracted from infiltrated tobacco leaves with protein extraction buffer, and then incubated with 20 μl anti-GFP agarose beads (AT0089) for 2 h at 4℃. Use the magnetic rack to absorb the supernatant, slowly add the collected GFP beads rotate centrifuge tube, mix well, use magnetic rack to absorb supernatant, repeat washing 5 times with wash buffer. The precipitated proteins were eluted with 3×SDS loading buffer at 95°C for 5 min. The samples were subjected to immunoblot analysis using the indicated antibodies. Primers used for the vector construction for Pull-down and Co-IP assay were presented in Supplementary Table 4.

Cell-free protein degradation assay

The experiments were conducted as described previously[8]. The total proteins of 10 DAF WT endosperm were extracted, and then 300ng of purified His-OsGBSSI or His-OsPPDKB protein was incubated with or without purified recombinant GST-OsHsp70-2, GST-oshsp70-2, GST-OsHsp40-1 and GST-oshsp40-1 protein in 200μg of extracted total protein in degradation buffer at 28°C for the individual assays. The protease inhibitor MG132 (Sigma-Aldrich) was used to suppress the degradation of His-OsGBSSI or His-OsPPDKB. Reactions were terminated at the indicated time points for determination of OsGBSSI or His-OsPPDKB abundance by western blotting using an anti-His-antibody.

Haplotype analysis

The haplotypes were analyzed based on the SNPs (frequencies >5%) identified within a full-length coding sequence of *OsHsp40-1*and *OsHsp70-2* from 3000 rice accessions (https://snp-seek.irri.org/_snp.zul). Haplotypic phenotype and genotype statistics and analysis of these two genes were further performed using 155 rice landraces collected from a wide geographic range of the world (Supplementary Table3). According to the nucleotide and amino acid sequence polymorphisms, *OsHsp40-1* contains about 13 haplotypes and can be grouped into 4 types, and *OsHsp70-2* contains about 14 haplotypes and can be grouped into 5 types.

References

1. Kang, H. G., Park, S., Matsuoka, M. & An, G. White-core endosperm floury endosperm-4 in rice is generated by knockout mutations in the C4-type pyruvate orthophosphate dikinase gene (*OsPPDKB*). *Plant J.* **42**, 901-911 (2005).

2. Peng, C. *et al. FLOURY ENDOSPERM6* encodes a CBM48 domain-containing protein involved in compound granule formation and starch synthesis in rice endosperm. *Plant J.* **77**, 917-930 (2014).

3. Li, S. F. *et al. OsBT1* encodes an ADP-glucose transporter involved in starch synthesis and

compound granule formation in rice endosperm. *Sci Rep*. **7**, 40124 (2017)

4. Shan, Q. W. *et al*. Targeted genome modification of crop plants using a CRISPR-Cas system. *Nat Biotechnol*. **31**, 686-688 (2013).
5. Zhang, Y. *et al*. A highly efficient rice green tissue protoplast system for transient gene expression and studying light/chloroplast-related processes. *Plant Methods*. **7**, 30 (2011).
6. Chen, S. B. *et al*. A highly efficient transient protoplast system for analyzing defence gene expression and protein-protein interactions in rice. *Mol Plant Pathol*. 7, 417-427 (2006).
7. Chen, H. M. *et al*. Firefly luciferase complementation imaging assay for protein-protein interactions in plants. *Plant Physiol*. **146**, 368-376 (2008).
8. Li, Z. Y. *et al*. The OsNAC23-Tre6P-SnRK1 a feed-forward loop regulates sugar homeostasis and grain yield in rice. *Mol. Plant*. **15**, 706-722 (2022).

Supplementary Figures:

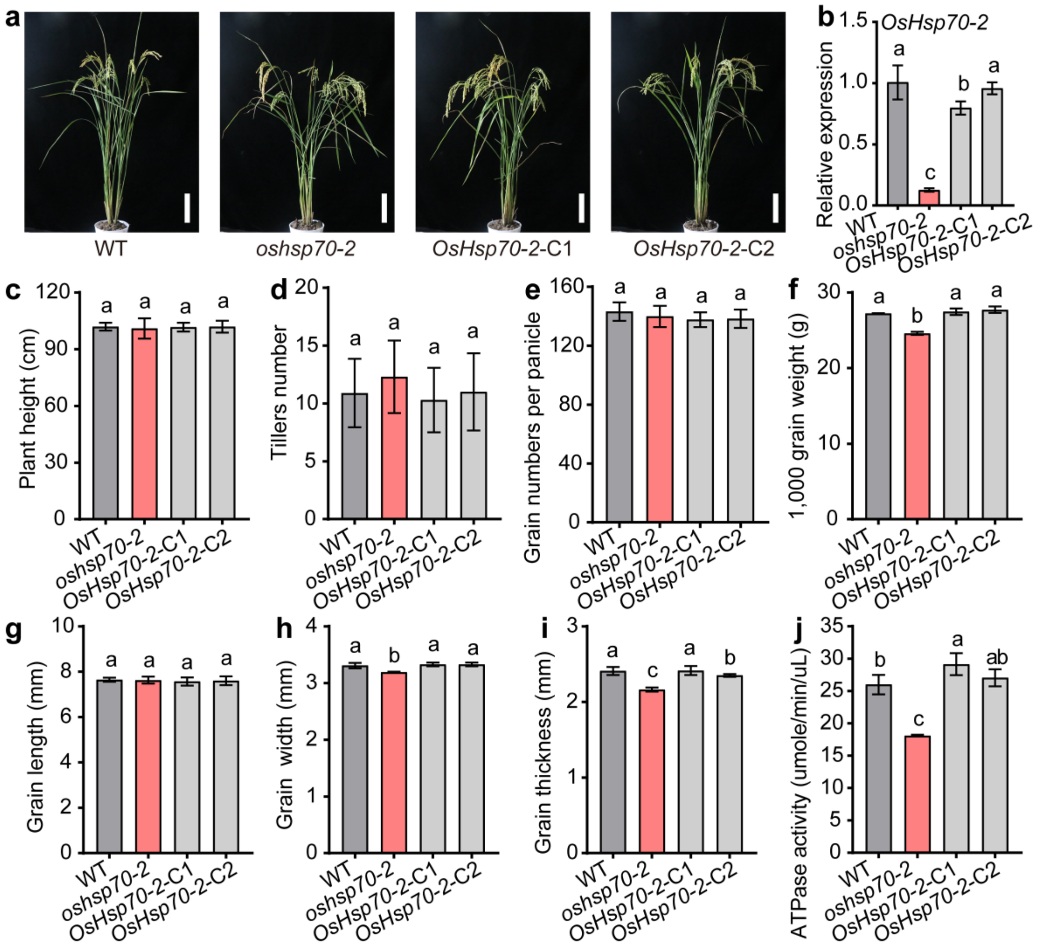

Supplementary Figure 1. Characteristics of wild-type (WT), *oshsp70-2* and complementation line plants.

(a) The plant phenotypes of WT, *oshsp70-2*, and complementation lines (*OsHsp70-2-C1* and *C2*). Scale bars, 10cm. **(b)** Relative expression levels of *OsHsp70-2* in WT, *oshsp70-2*, and complementation lines. **(c-i)** The plant height (c), tillers number (d), grain number per panicle (e), 1000 grain weight (f), grain length (g), grain width (h), and grain thickness (i) of WT, *oshsp70-2*, and complementation lines. **(j)** The total ATPase enzyme activity in developing endosperm of WT, *oshsp70-2*, and complementation lines. All plants were grown in natural high temperature conditions in Hangzhou, 2020 (daily average temperature 30℃ during grain-filling stage). Data in (b-j) are means ± SD, n=10 in (c-e, g-i), n=3 in (b, f, j), and no less than 200 grains per replication in (f). Different letters indicate significant difference at $p < 0.05$ by ANOVA and Duncan's test.

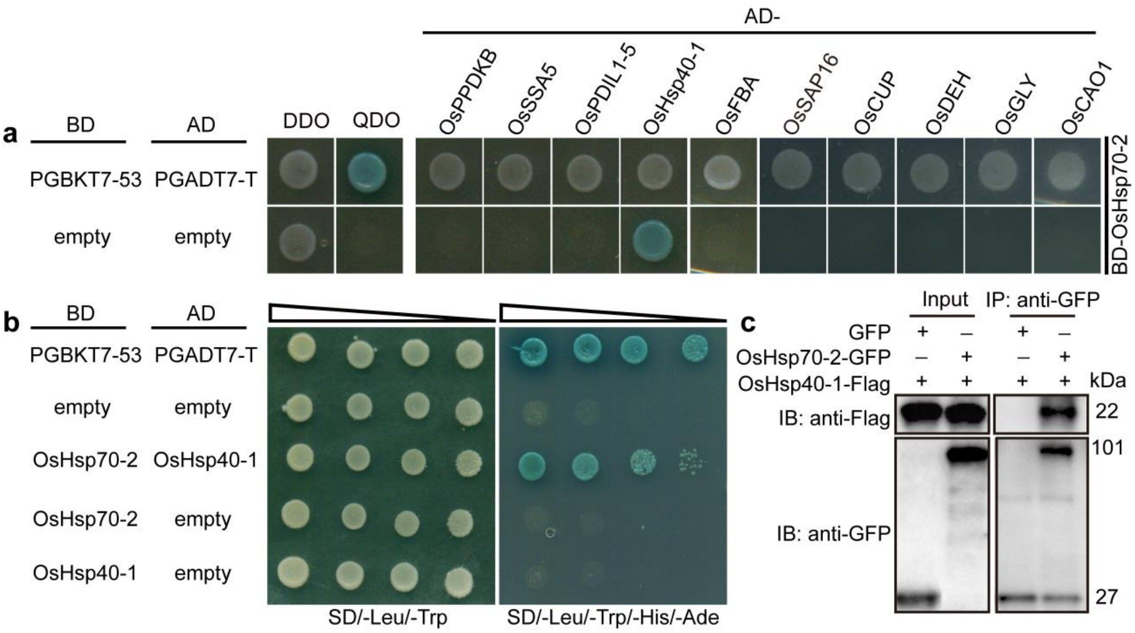

Supplementary Figure 2. OsHsp70-2 interact with OsHsp40-1.

(a-b) The interaction between OsHsp70-2 and OsHsp40-1 was detected by Yeast two-hybrid (Y2H) assays, Y2H screening library showed OsHsp70-2 interact with OsHsp40-1 (a), and there was no self-activation in both OsHsp70-2 and OsHsp40-1 (b). OsHsp70-2 and OsHsp40-1 were fused to activation domain (AD) or binding domain (BD). “pGBKT7-53+ pGADT7-T” was the positive control, empty “pGBKT7+ pGADT7” was the negative control, and yeast cells expressing the indicated proteins were plated onto nonselective medium (SD/-Leu/-Trp, DDO) or selective medium (SD/-Leu/-Trp/-Ade/ -His, QDO). **(c)** The interaction between OsHsp70-2 and OsHsp40-1 was detected by the Co-IP assays in *N. benthamiana* leaves.

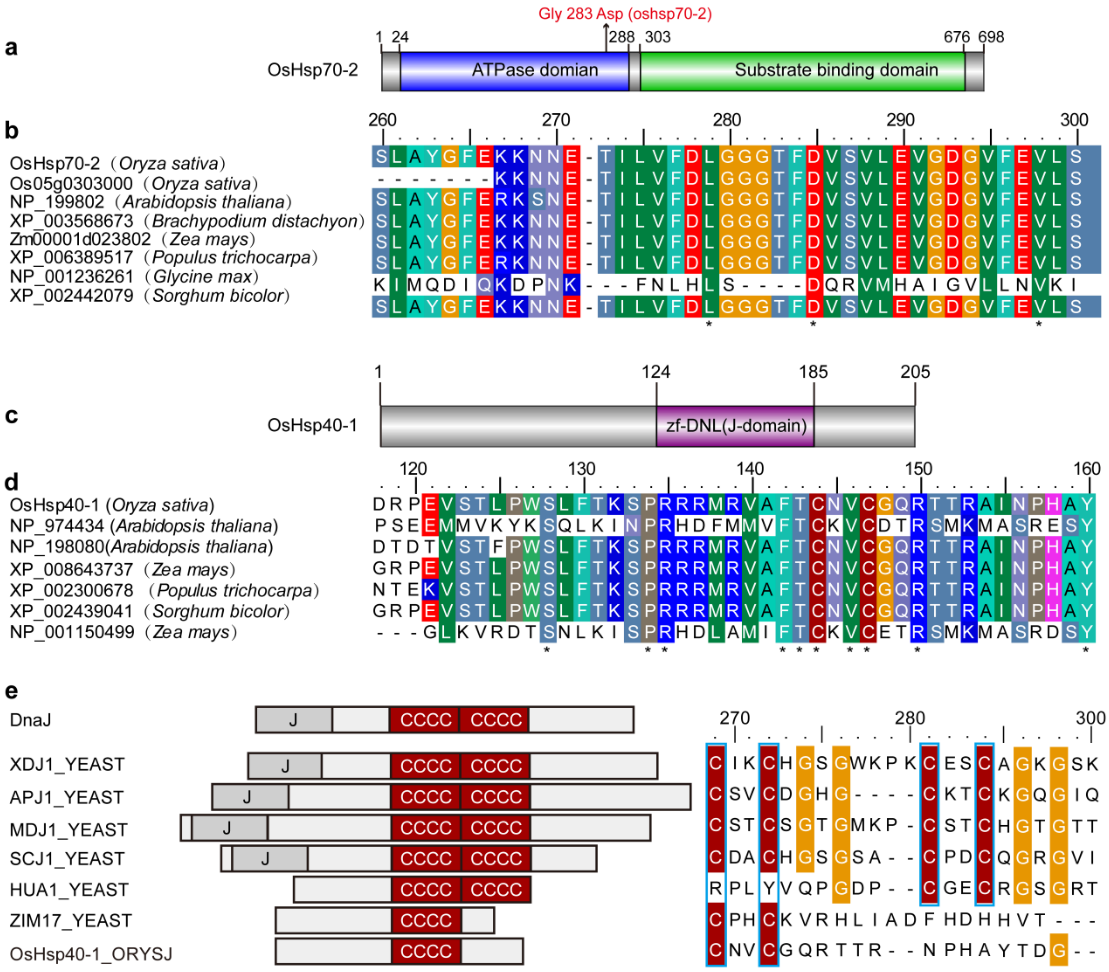

Supplementary Figure 3. The conserved motifs in OsHsp70-2 and OsHsp40-1.

(a) The main domains of OsHsp70-2. The OsHsp70-2 including an ATPase domain in N terminal and a C-terminal substrate binding domain. An amino acid conversion from glycine (Gly) to aspartic acid (Asp) at position 283 in *oshsp70-2* mutant is indicates by an arrow. **(b)** Sequence alignment of OsHsp70-2 and some homolog proteins in higher plant. The asterisks represent the core amino acids. **(c)** The main domains of OsHsp40-1. **(d)** Sequence alignment of OsHsp40-1 homologies from *Arabidopsis thaliana*, *Zea mays*, *Populus trichocarpa*, and *Sorghum bicolor*. Using ClustalX alignment amino acid sequences, the conserved regions are marked with asterisks. **(e)** OsHsp40-1 is a DnaJ-Type zinc finger Protein. Schematic (left) represents the classical J-proteins from yeast. It revealed sequence similarity between the zinc finger domains of four type I J-proteins and the novel proteins HUA1, ZIM17 and OsHsp40-1. Both ZIM17 and OsHsp40-1 align with the central region of the J-proteins. Sequence alignment of zinc finger domain the OsHsp40-1 and other homolog proteins in yeast (right).

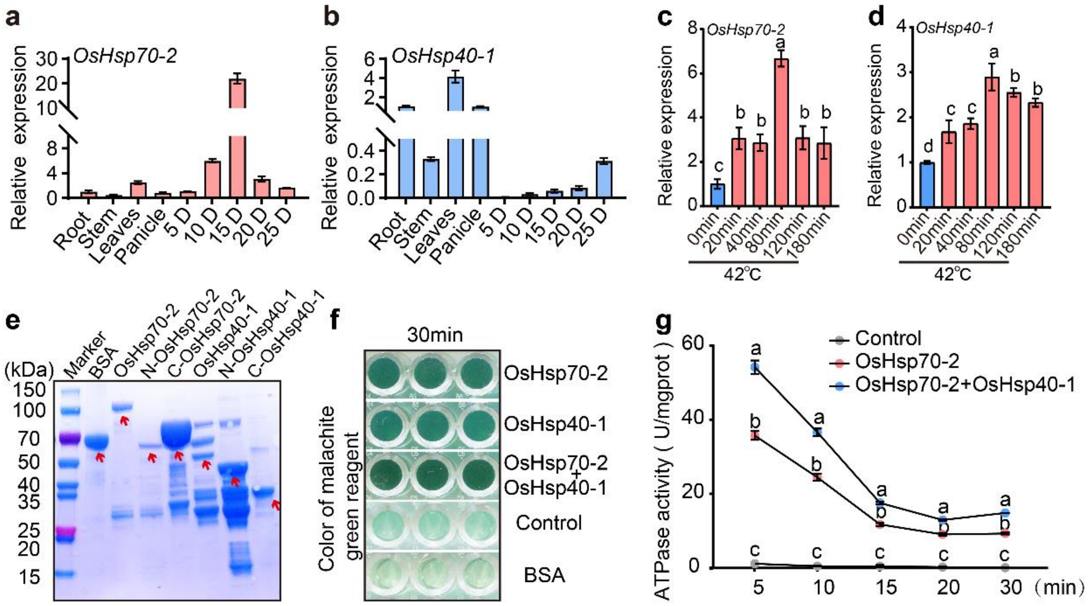

Supplementary Figure 4. The expression patterns, ATPase activity of the OsHsp70-2 and OsHsp40-1.

(a-b) Expression levels of *OsHsp70-2* (a) and *OsHsp40-1* (b) in different tissues of rice. *Actin1* gene (*Os03g0718150*) was used as internal control. D, the developing grains of different days after fertilization. **(c-d)** Relative expression level of *OsHsp70-2* and *OsHsp40-1* in two-week-old seedlings under heat stress treatment (42°C). **(e)** SDS-PAGE electrophoresis of the GST- fusion prokaryotic expression proteins of the full-length and truncated OsHsp70-2 and OsHsp40-1. **(f)** ATPase activity assay. ATPase and GTPase catalyze ATP and GTP to release ADP or GDP. The released inorganic phosphorus ions form stable dark green inorganic phosphorus ions with malachite green reagent. The ultraviolet spectrophotometer is set at 620nm, and then the absorbance is determined at this wavelength. (**g**) Determination ATP enzyme activity of the purified proteins after different reaction times. The plants in (a,b) were grown in natural high temperature conditions in Hangzhou, 2021 (daily average temperature 28.4℃ during grain-filling stage). Data in (a-d, g) are means ± SD from three biological replicates.

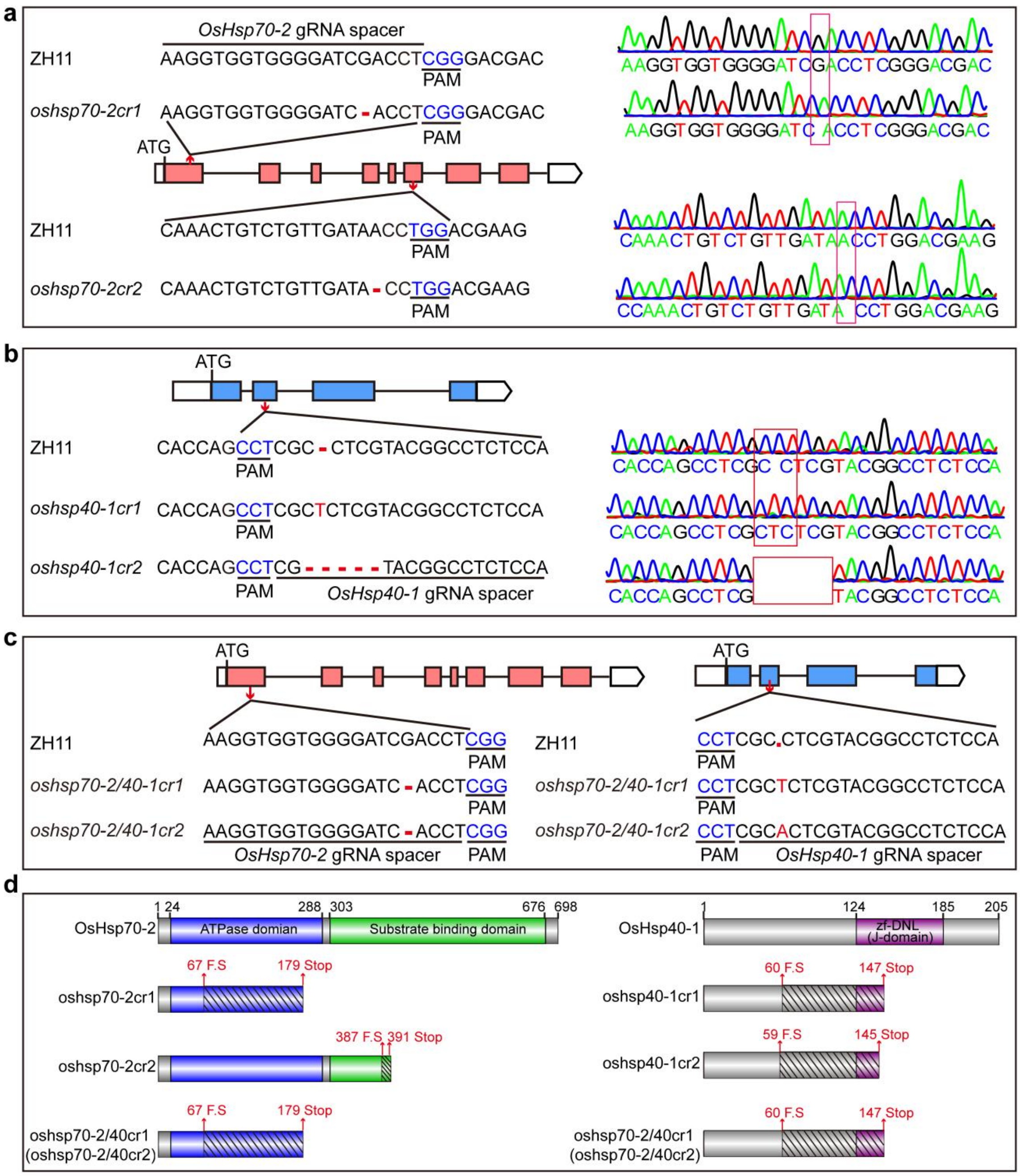

Supplementary Figure 5. The single and double mutants of *OsHsp70-2* and *OsHsp40-1* were obtained by CRISPR/Cas9 gene editing in the Zhonghua11 background.

(a) Diagram of the *OsHsp70-2* gene and the two mutations (*oshsp70-2cr1*, *cr2*) obtained by CRISPR-Cas9. **(b)** Diagram of the *OsHsp40-1* gene and the two mutations (*oshsp40-1cr1*, *cr2*) obtained by CRISPR-Cas9. **(c)** The two double mutants of *OsHsp70-2* and *OsHsp40-1* obtained by CRISPR-Cas9. **(d)** The amino acids variations in the single and double mutants of *OsHsp70-2* and *OsHsp40-1*. The locations of frameshift (F.S.) and premature terminations (Stop) are shown with red numbers. In (a-c) The red arrow at the bottom of the structure diagram indicates the mutation site; the PAM sequence is underlined with blue font; the red word indicates the insertion base, and the red "-" indicates the deletion base.

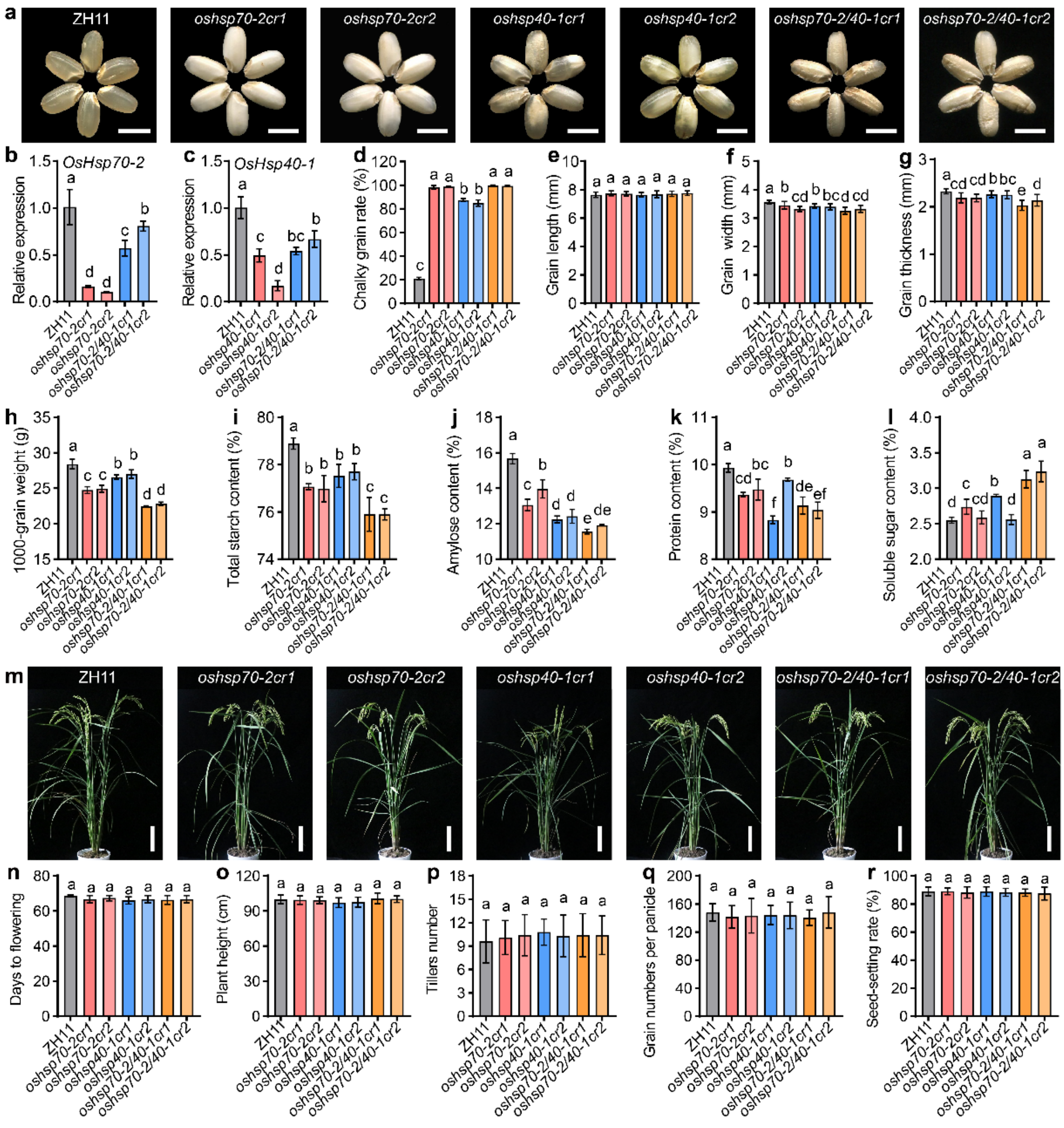

Supplementary Figure 6. *OsHsp70-2* and *OsHsp40-1* modulate rice quality and grain yield under natural high temperature conditions.

(a) Grain appearance of ZH11 (WT) and single and double mutants of *OsHsp70-2* and *OsHsp40-1*. Scale bars, 5 mm. **(b-c)** The relative expression levels of *OsHsp70-2* and *OsHsp40-1* in the 10 DAF endosperm of ZH11, the single and double mutants. **(d-h)** Chalky grain rate (d), grain length (e), grain width (f), grain thickness (g), and 1000-grain weight (h) of ZH11, the single and double mutants. (**i-l**) The contents of total starch (i), amylose (j), protein (k), and soluble sugar (l) in endosperm of ZH11, the single and double mutants. **(m)** The plant phenotypes of ZH11, the single and double mutants. (**n-r**) The important agronomic traits of ZH11, the single and double mutants. All plants were grown in natural high temperature conditions in Hangzhou, 2021 (daily average temperature 28.4℃ during grain-filling stage). Data in (b-l, n-r) are means ± SD, n=10 in (e-g, n-r), n=3 in (b-d, h, i-l), and no less than 200 grains per replication in (d, h). Different letters indicate significant difference at $p < 0.05$ by ANOVA and Duncan's test.

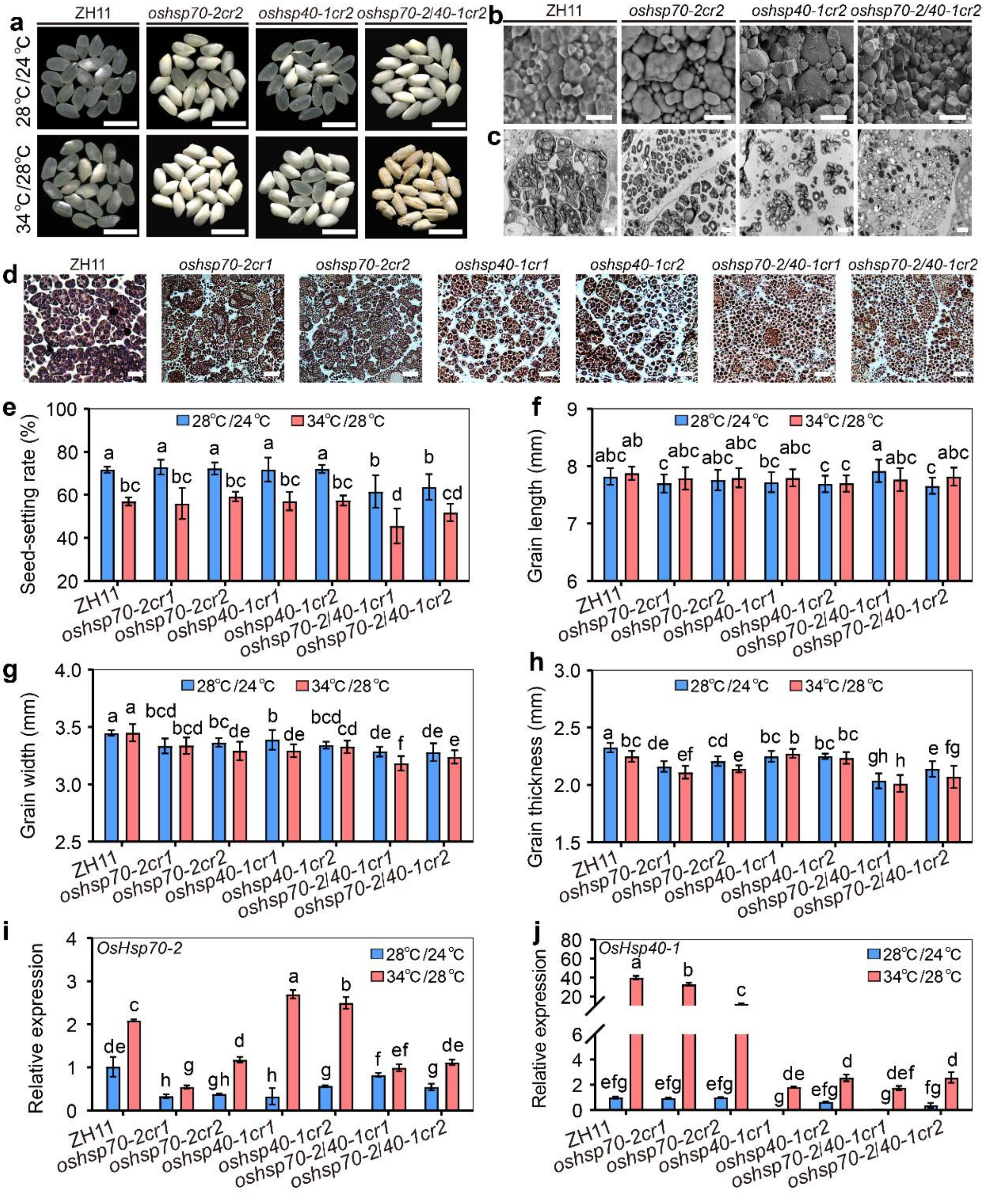

Supplementary Figure 7. Characterization of the single and double mutants of *OsHsp70-2* and *OsHsp40-1* under high- and normal-temperature treatments.

(a) Appearance of milled rice of ZH11 (WT), the single and double mutants under artificial high-temperature (HT, 34°C, 12h light/28°C, 12h dark) and normal-temperature treatments (NT, 28°C, 12h light/24°C, 12h dark) during grain filling stage. Scale bars, 10 mm. **(b)** Scanning electron microscopy of the central area of mature grain endosperm of WT and mutants under HT, Scale bars, 10 μm. **(c-d)** Transmission electron microscopy (c) and semi-thin sections (d) of central region of 9DAF endosperm of WT and mutants under HT. Scale bars, 2μm in (c), 50μm in (d). **(e-h)** Seed setting rate (e), grain length (f), grain width (g) and grain thickness (h) of WT and mutants under HT and NT conditions. (**i-j**) Relative expression level of *OsHsp70-2* and *OsHsp40-1* in WT, *oshsp70-2cr1*, *oshsp40-1cr1* and

oshsp70-2/40-1cr1 under NT and HT during grain filling stage. Data in (e-j) are means ± SD, *n*=10 in (f-h), *n*=4 in (e), and n=3 in (i-j). Different letters indicate significant difference at $p < 0.05$ by ANOVA and Duncan's test.

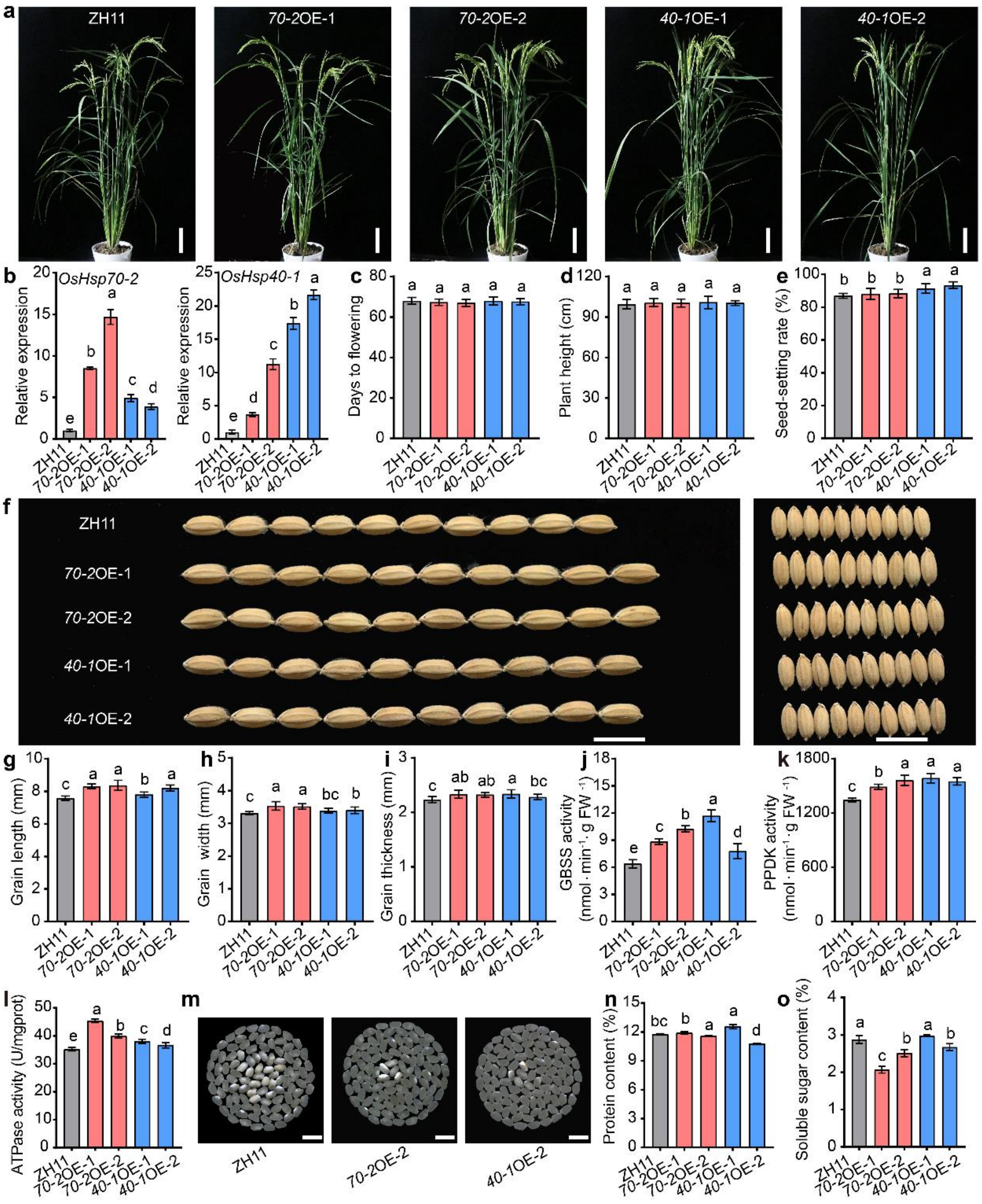

Supplementary Figure 8. Overexpression of *OsHsp70-2* and *OsHsp40-1* under the background of ZH11.

(**a**) The plants phenotypes of ZH11 and *OsHsp70-2*, *OsHsp40-1* OE lines. Scale bars, 10cm. **(b)** The relative expression levels of *OsHsp70-2* and *OsHsp40-1* in ZH11 and OE lines. (**c-e**) Days to flowering (c), plant height (d), and seed setting rate (e) of the ZH11and OE lines. **(f)** Grain size of ZH11 and OE lines. Scale bars, 10mm. **(g-i)** Grain length (g), grain width (h), and grain thickness (i) of ZH11, and OE lines. **(j-l)** The activity of GBSS, PPDK and ATP in 10 DAF endosperm of ZH11 and OE lines. **(m)** The appearance of milled rice of ZH11 and OE lines. Scale bars, 10mm. **(n-o)** The percent content of protein (n) and soluble sugar (o) in endosperm of ZH11 and OE lines. All plants were grown in natural high temperature conditions in Hangzhou, 2021 (daily average temperature 28.4℃ during grain-filling stage). Data are means ± SD, n=10 in (c-e, g-i), n=3 in (b, j-l, n-o). Different letters indicate significant difference at $p < 0.05$ by ANOVA and Duncan's test.

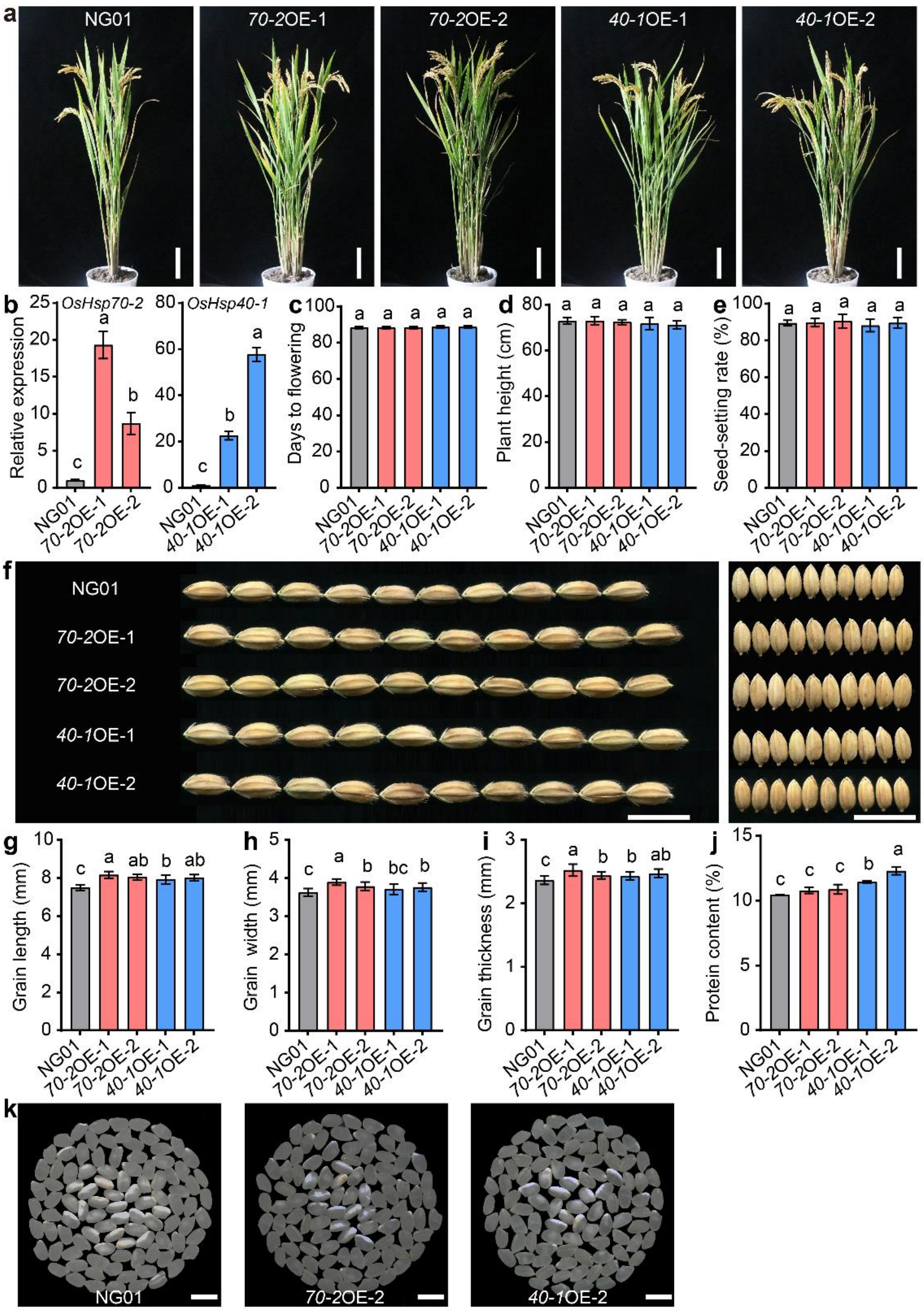

Supplementary Figure 9. Overexpression of *OsHsp70-2* and *OsHsp40-1* under the background of *japonica* rice Ninggeng01.

(a) The plants phenotypes of Ninggeng01 (NG01) and *OsHsp70-2*, *OsHsp40-1* OE lines. Scale bars, 10cm. **(b)** The relative expression levels of *OsHsp70-2* and *OsHsp40-1* in NG01 and OE lines. **(c-e)** Days to flowering (c), plant height (d), and seed setting rate (e) of the NG01 and OE lines. **(f)** Grain size of NG01 and OE lines. Scale bars, 10mm. **(g-j)** Grain length (g), grain width (h), grain thickness (i), and the protein content in endosperm (j) of NG01 and OE lines. **(k)** The appearance of milled rice of NG01 and OE lines. Scale bars, 10mm. All plants were grown in natural high temperature conditions in Hangzhou, 2021 (daily average temperature 28.4℃ during grain-filling stage). Data are means ± SD, n=10 in (c-e, g-i), n=3 in (b, j). Different letters indicate significant difference at $p < 0.05$ by ANOVA and Duncan's test.

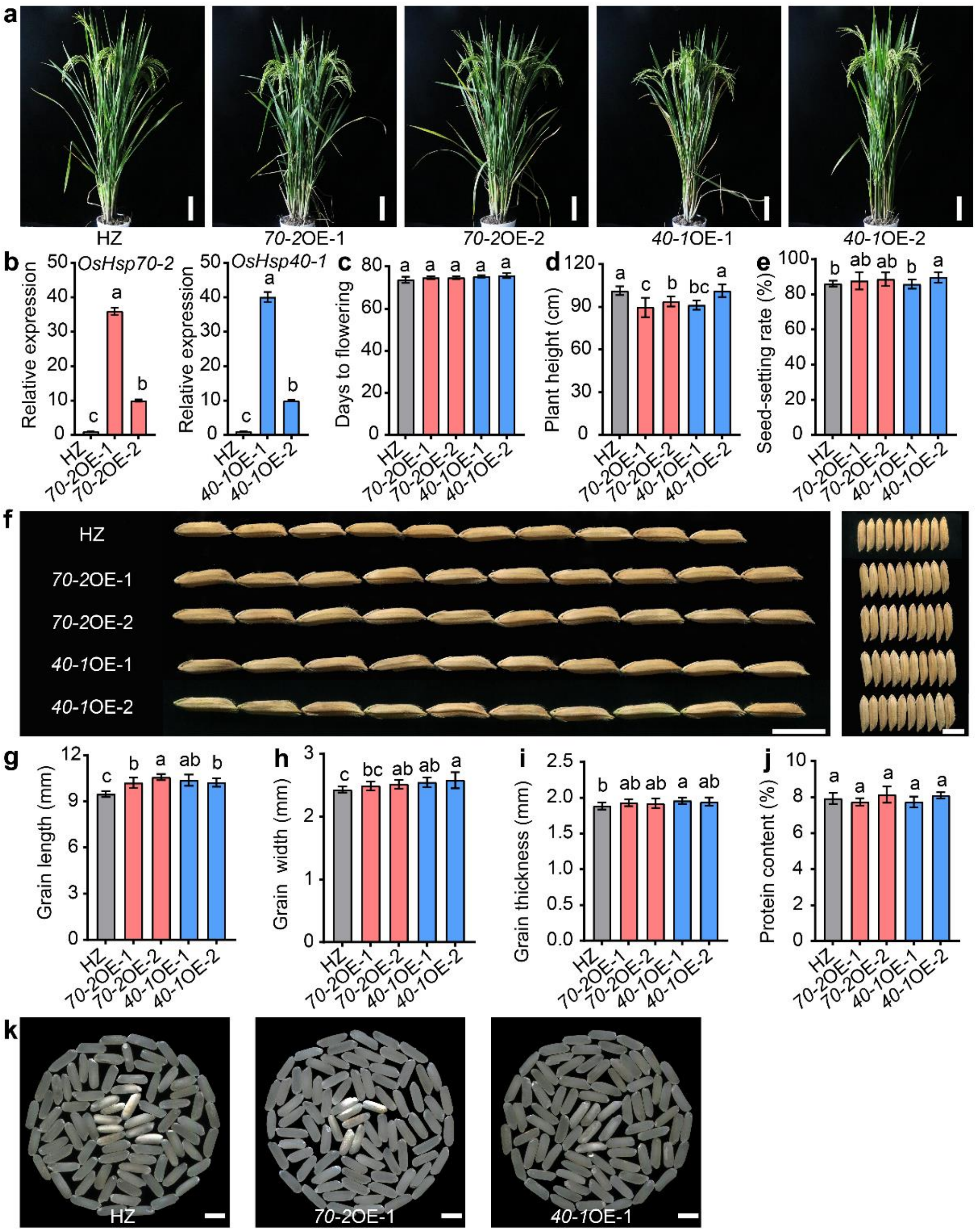

Supplementary Figure 10. Overexpression of *OsHsp70-2* and *OsHsp40-1* under the background of *indica* rice Huazhan.

(a) The plants phenotypes of Huazhan (HZ) and *OsHsp70-2*, *OsHsp40-1* OE lines. Scale bars, 10cm. **(b)** The relative expression levels of *OsHsp70-2* and *OsHsp40-1* in HZ and OE lines. **(c-e)** Days to flowering (c), plant height (d), and seed setting rate (e) of the HZ and OE lines. **(f)** Grain size of HZ and OE lines. Scale bars, 10mm. **(g-j)** Grain length (g), grain width (h), grain thickness (i), and the protein content in endosperm (j) of HZ and OE lines. **(k)** The appearance of milled rice of HZ and OE lines. Scale bars, 10mm. All plants were grown in natural high temperature conditions in Hangzhou, 2021 (daily average temperature 28.4°C during grain-filling stage). Data are means ± SD, n=10 in (c-e, g-i), n=3 in (b, j). Different letters indicate significant difference at $p < 0.05$ by ANOVA and Duncan's test.

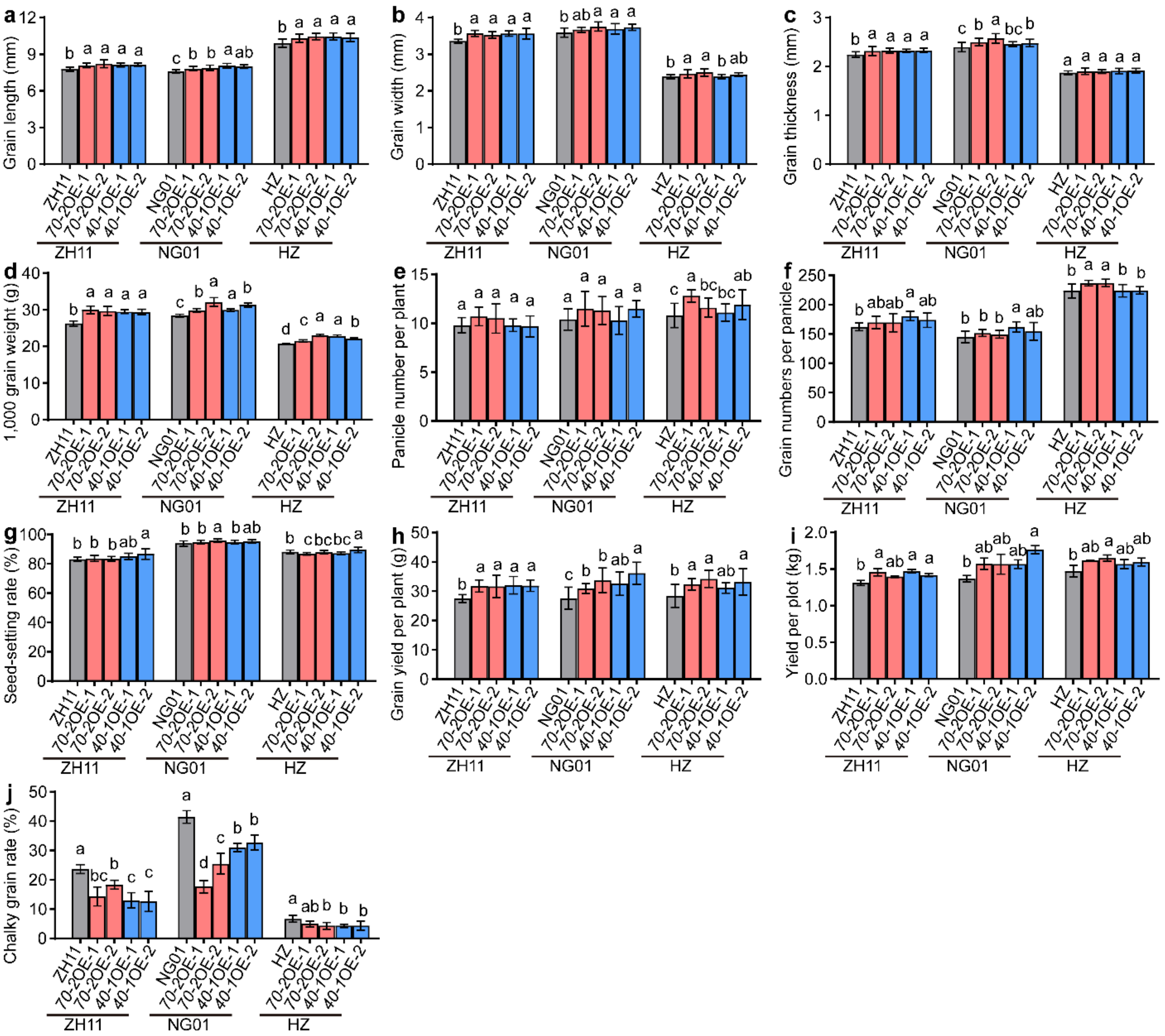

Supplementary Figure 11. Overexpression of *OsHsp70-2* and *OsHsp40-1* improve rice grain yield under natural high temperature conditions in Hangzhou, 2023.

(a-c) The grain length (a), grain width (b), and grain thickness (c) of *OsHsp70-2* and *OsHsp40-1* OE lines under the background of ZH11, NingGeng01(NG01), and Huazhan (HZ). **(d-i)** The thousand grain weight (d), panicle number per plant (e), grain number per panicle (f), seed setting rate (g), grain yield per plant (h), and grain yield per plot (i) of OE lines of *OsHsp70-2* and *OsHsp40-1* under background of ZH11, NG01, and HZ. **(j)** The chalky grain ratio of OE lines under three genetic backgrounds. All plants were grown in natural high temperature conditions in Hangzhou, 2023 (daily average temperature 29.2℃ during grain-filling stage). Data are means ± SD, n=10 in (a-c, e-h), n=3 in (d, j), and n=2 in (i), and no less than 200 grains per replication in (d, j). Different letters indicate significant difference at $p < 0.05$ by ANOVA and Duncan's test.

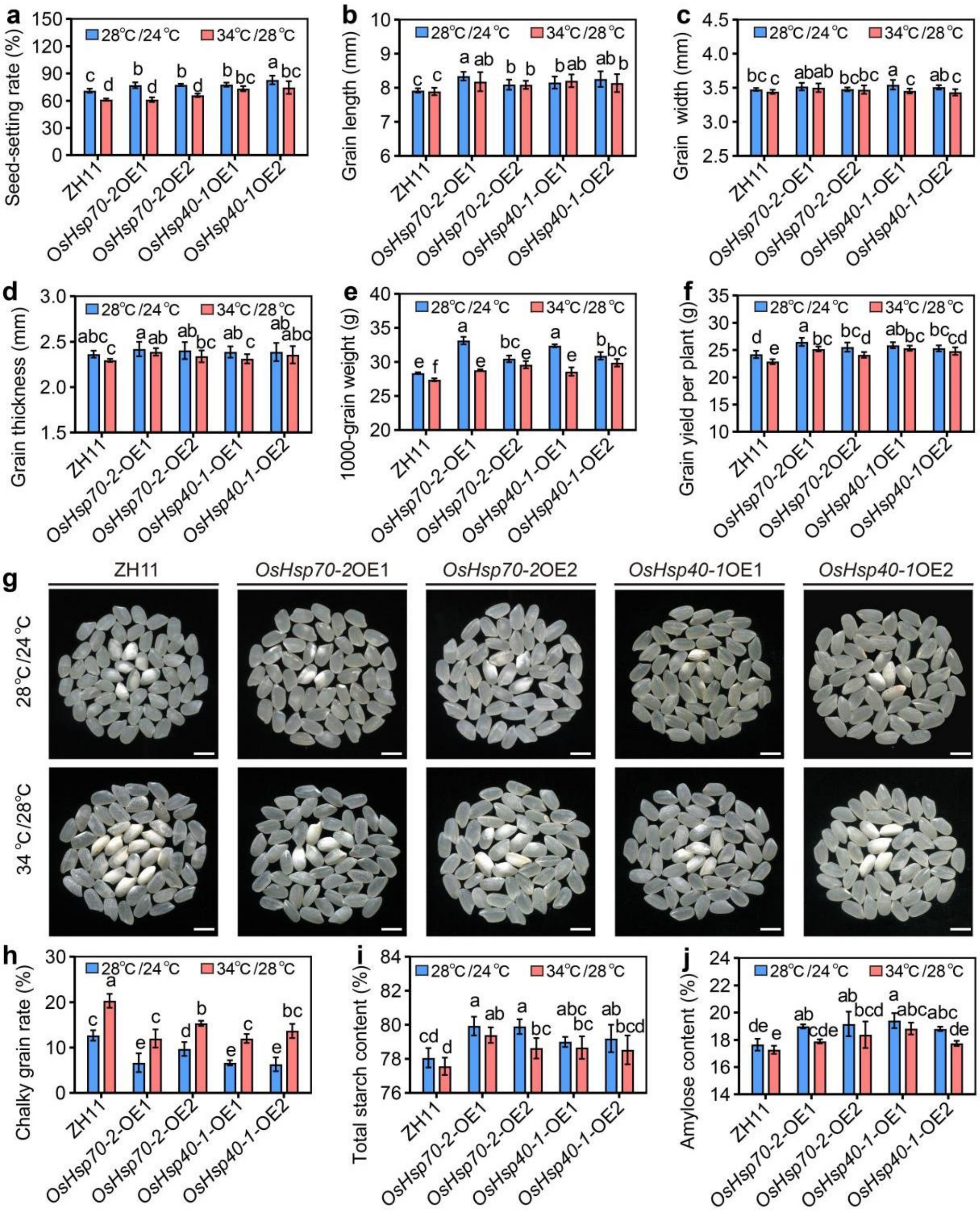

Supplementary Figure 12. Characterization of the overexpression lines of *OsHsp70-2* and *OsHsp40-1* in ZH11 backgrounds under normal- and high-temperature conditions.

(a-f) The seed setting rate (a), grain length (b), grain width (c), grain thickness (d), 1000-grain weight (e), and grain yield per plant (f) of ZH11 and the overexpression (OE) lines of *OsHsp70-2* and *OsHsp40-1* under artificial high-temperature (HT, 34°C, 12h light/28°C, 12h dark) and normal-temperature conditions (NT, 28°C, 12h light/24°C, 12h dark) during grain filling stage. **(g)** Appearance of milled rice of ZH11, the OE lines of *OsHsp70-2* and *OsHsp40-1* under HT and NT conditions. Scale bars, 5 mm. **(h-j)** The chalky grain rate (h), total starch content (i), and amylose content (j) of endosperm of ZH11 and OE lines of *OsHsp70-2* and *OsHsp40-1* under NT and HT conditions. Data in (a-f, h-j) are means ± SD, n=4 in (a, f), n=10 in (b-d), n=3 in (e, h-j) and no less than 200 grains per replication in (e, h). Different letters indicate significant difference at p < 0.05 by ANOVA and Duncan's test.

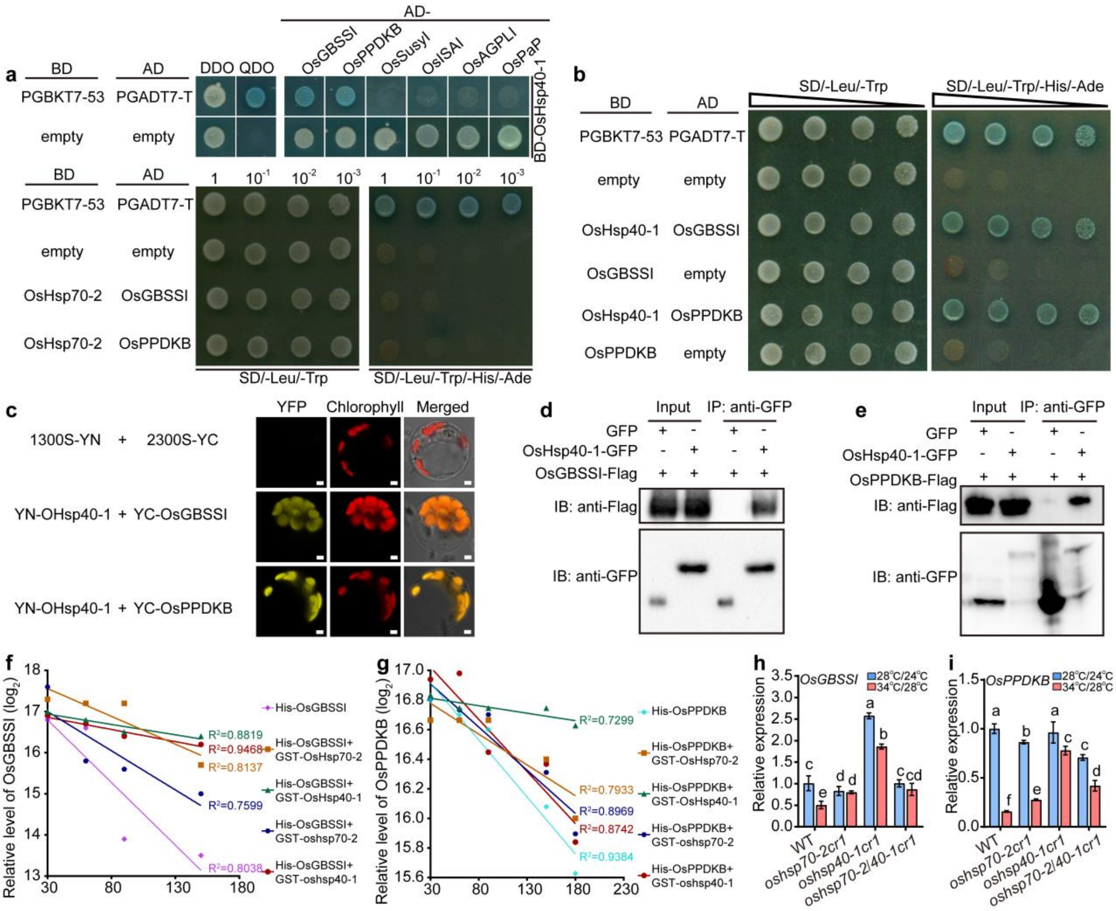

Supplementary Figure 13. OsHsp40-1 interacts with OsGBSSI and OsPPDKB.

(a-b) Yeast two-hybrid (Y2H) assays showed that OsHsp40-1 rather than OsHsp70-2 interact with OsGBSSI and OsPPDKB. OsHsp40-1 and OsHsp70-2 were fused to binding domain (BD), and OsGBSSI and OsPPDKB were fused to activation domain (AD). “pGBKT7-53+ pGADT7-T” was the positive control, empty “pGBKT7+ pGADT7” was the negative control, and yeast cells expressing the indicated proteins were plated onto nonselective medium (SD/-Leu/-Trp, DDO) or selective medium (SD/-Leu/-Trp/-Ade/-His, QDO). **(c**) BiFC assays verified the interaction between OsHsp40-1 and OsGBSSI or OsPPDKB, 10μm. **(d-e)** Interaction between OsHsp40-1 and OsGBSSI or OsPPDKB detected by CoIP assays. **(f-g)** Degradation curve of His-OsGBSSI (f) and His-OsPPDKB (g) in the absence or presence of OsHsp70-2 and OsHsp40-1. Protein intensities were quantified using the Image J software. **(h-i)** The relative expression level of *OsPPDKB* and *OsGBSSI* in wild type, *oshsp70-2cr1*, *oshsp40-1cr1* and *oshsp70-2/40-1cr1* under artificial high-temperature (34°C, 12h light/28°C, 12h dark) and normal-temperature treatments (28°C, 12h light/24°C, 12h dark) during grain filling stage. Values are means ± SD from three biological replicates. Different letters indicate significant difference at $p < 0.05$ by ANOVA and Duncan’s test.

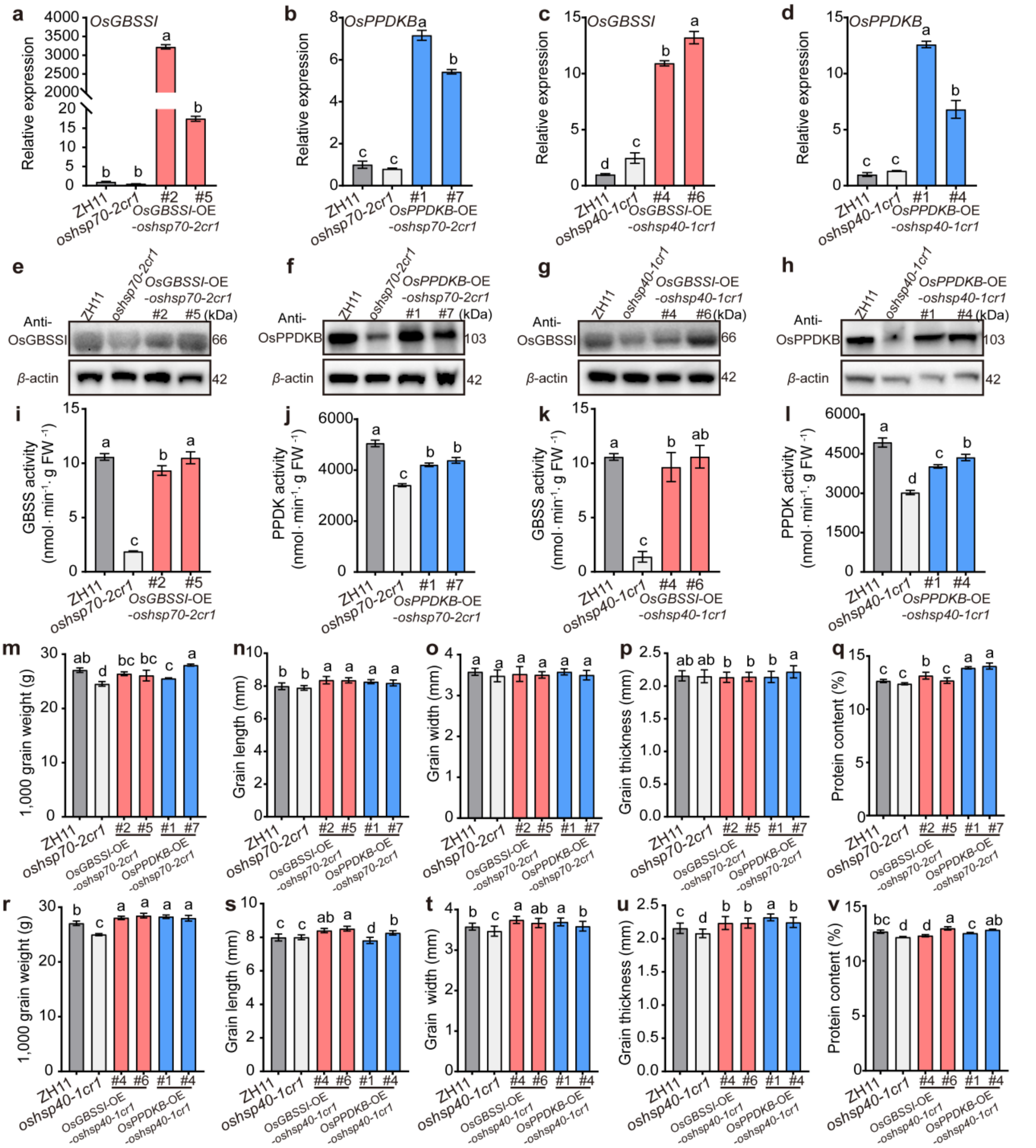

Supplementary Figure 14. Overexpression of *OsGBSSI* and *OsPPDKB* in the backgrounds of *oshsp70-2cr1* and *oshsp40-1cr1*, respectively.

(a-d) The relative expression levels of *OsGBSSI* and *OsPPDKB* in endosperm of WT (ZH11), mutants, and OE plants. **(e-h)** Immunoblot analysis of the protein levels of OsGBSSI and OsPPDKB in the endosperm of WT, mutants, and OE plants. **(i-l)** The GBSS and PPDK enzyme activities detected in the endosperm of WT, mutants, and OE plants. **(m-v)** The 1000 grain weight (m, r), grain length (n, s), grain width (o, t), grain thickness (p, u) and protein content (q, v) of WT, mutants, and OE plants. All plants were grown in natural high temperature conditions in Hangzhou, 2022 (daily average temperature 31.2℃ during grain-filling stage). Data are means ± SD, *n*=10 in (n-p, s-u), *n*=3 in (a-d, i-m, r, q, v), and no less than 200 grains per replication in (m, r). Different letters indicate significant difference at $p < 0.05$ by ANOVA and Duncan’s test.

a

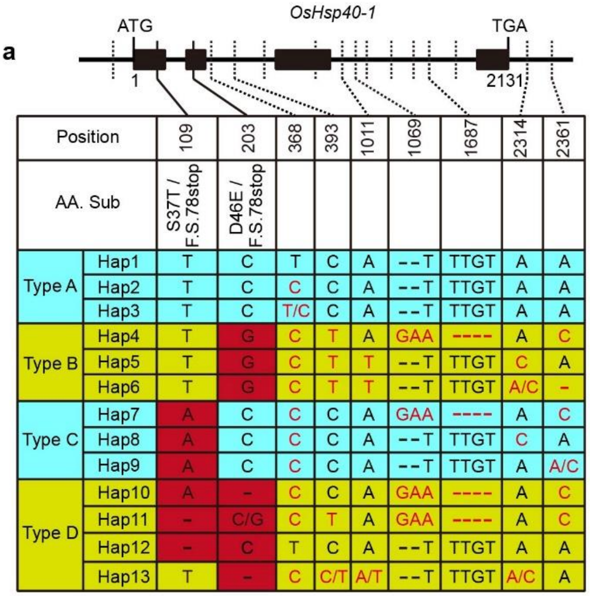

Position		109	203	368	393	1011	1069	1687	2314	2361	TEJ	TRJ	AUS	IND	ARO	O.Ruf	All
AA. Sub		S37T / F.S.78stop	D46E / F.S.78stop								*No.of accessions*						
Type A	Hap1	T	C	T	C	A	--T	TTGT	A	A	232	54	0	8	0	111	405
	Hap2	T	C	C	C	A	--T	TTGT	A	A	36	177	0	18	31	0	262
	Hap3	T	C	T/C	C	A	--T	TTGT	A	A	4	0	0	0	0	0	4
Type B	Hap4	T	G	C	T	A	GAA	----	A	C	0	0	1	120	0	2	123
	Hap5	T	G	C	T	T	--T	TTGT	C	A	20	89	97	279	0	23	508
	Hap6	T	G	C	T	T	--T	TTGT	A/C	-	0	0	2	0	0	0	2
Type C	Hap7	A	C	C	C	A	GAA	----	A	C	7	1	75	720	10	106	919
	Hap8	A	C	C	C	A	--T	TTGT	C	A	4	154	1	331	19	6	515
	Hap9	A	C	C	C	A	--T	TTGT	A	A/C	0	0	1	14	0	0	15
Type D	Hap10	A	-	C	C	A	GAA	----	A	C	0	0	2	8	0	0	10
	Hap11	-	C/G	C	T	A	GAA	----	A	C	0	0	0	1	0	0	1
	Hap12	-	C	T	C	A	--T	TTGT	A	A	0	2	0	1	0	56	59
	Hap13	T	-	C	C/T	A/T	--T	TTGT	A/C	A	0	0	0	1	0	0	1

b

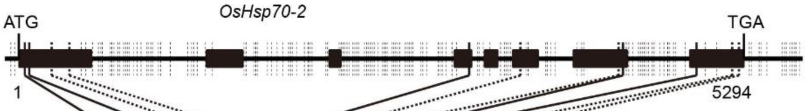

Position		61	70	222	345	3293	3663	4409	4413	4921	5249	5282	TEJ	TRJ	AUS	IND	ARO	O.Ruf	All
AA. Sub		P21A / F.S.179stop	A24S / F.S.187stop			S331T / F.S.340stop			A558S	K575R			*No.of accessions*						
Type A	Hap1	C	G	G	G	T	C	C	G	A	G	T	199	56	15	611	14	0	895
	Hap2	C	G	G	G	T	C	T	G	A	G	T	0	10	3	660	20	0	693
	Hap3	C	G	G	C	T	C	C	G	A	G	T	63	332	1	14	6	0	416
Type B	Hap4	G	G	G	G	T	C	C	G	A	G	T	1	2	64	38	1	0	106
	Hap5	G	G	G	-	T	C	C	G	A	G	T	0	0	7	0	0	0	7
Type C	Hap6	C	T	C	C	A	T	C	G	G	A	C	0	1	30	7	13	0	51
	Hap7	C	T	C	-	A	T	C	G	G	A	C	0	0	4	0	2	0	6
	Hap8	C	T	-	C	A	T	C	G	G	A	C	0	0	3	1	0	0	4
Type D	Hap9	C	G	G	G	T	C	T	T	A	G	T	0	0	0	45	0	0	45
Type E	Hap10	-	G	G	G	T	C	C	G	A	G	T	3	2	27	42	3	0	77
	Hap11	C	-	G	G	T	C	T	G	A	G	T	4	9	7	37	5	0	62
	Hap12	-	-	G	G	T	C	C	G	A	G	T	19	32	18	88	6	0	163
	Hap13	-	-	G	G	T	C	C	-	A	G	T	0	0	0	1	0	289	290
	Hap14	C	G	G	G	-	C	C	G	A	G	T	1	0	1	5	0	0	7

c

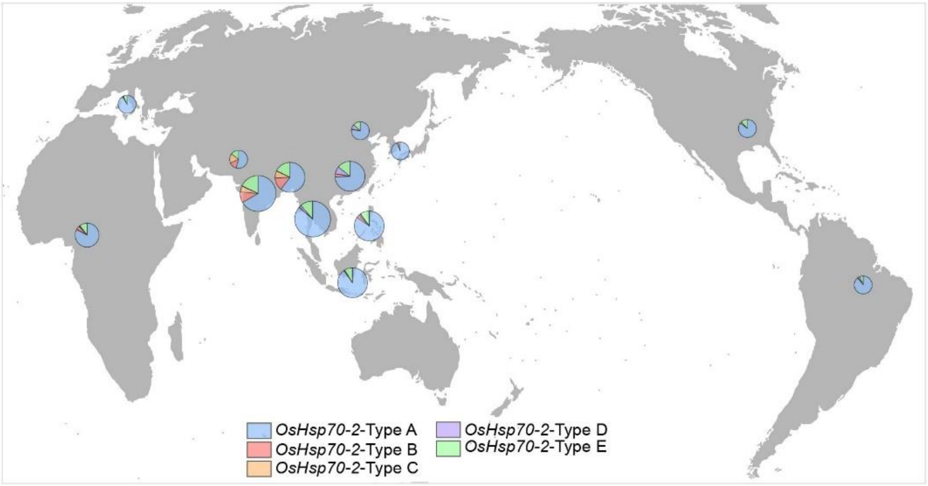

410 **Supplementary Figure 15. Haplotype analysis of *OsHsp70-2* and *OsHsp40-1*.**

(a-b) Haplotype analysis of *OsHsp40-1* and *OsHsp70-2* in ~3000 rice landraces and ~300 *O. rufipogon* accessions based on public resequencing data. *OsHsp40-1* contains about 13 haplotypes and can be grouped into 4 types (40-TypeA, B, C, and D) according to the nucleotide and amino acid sequence polymorphisms (a). *OsHsp70-2* contains about 14 haplotypes and can be grouped into 5 types (70-TypeA, B, C, D, and E) according to the nucleotide and amino acid sequence polymorphisms (b). *TEJ*, temperate *japonica*; *TRJ*, tropical *japonica*; *AUS*, *AUS* population; *IND*, *indica*; and *ARO*, aromatic population. **(c)** Geographic distributions of ~3000 rice landraces carrying different types of *OsHsp70-2* alleles.

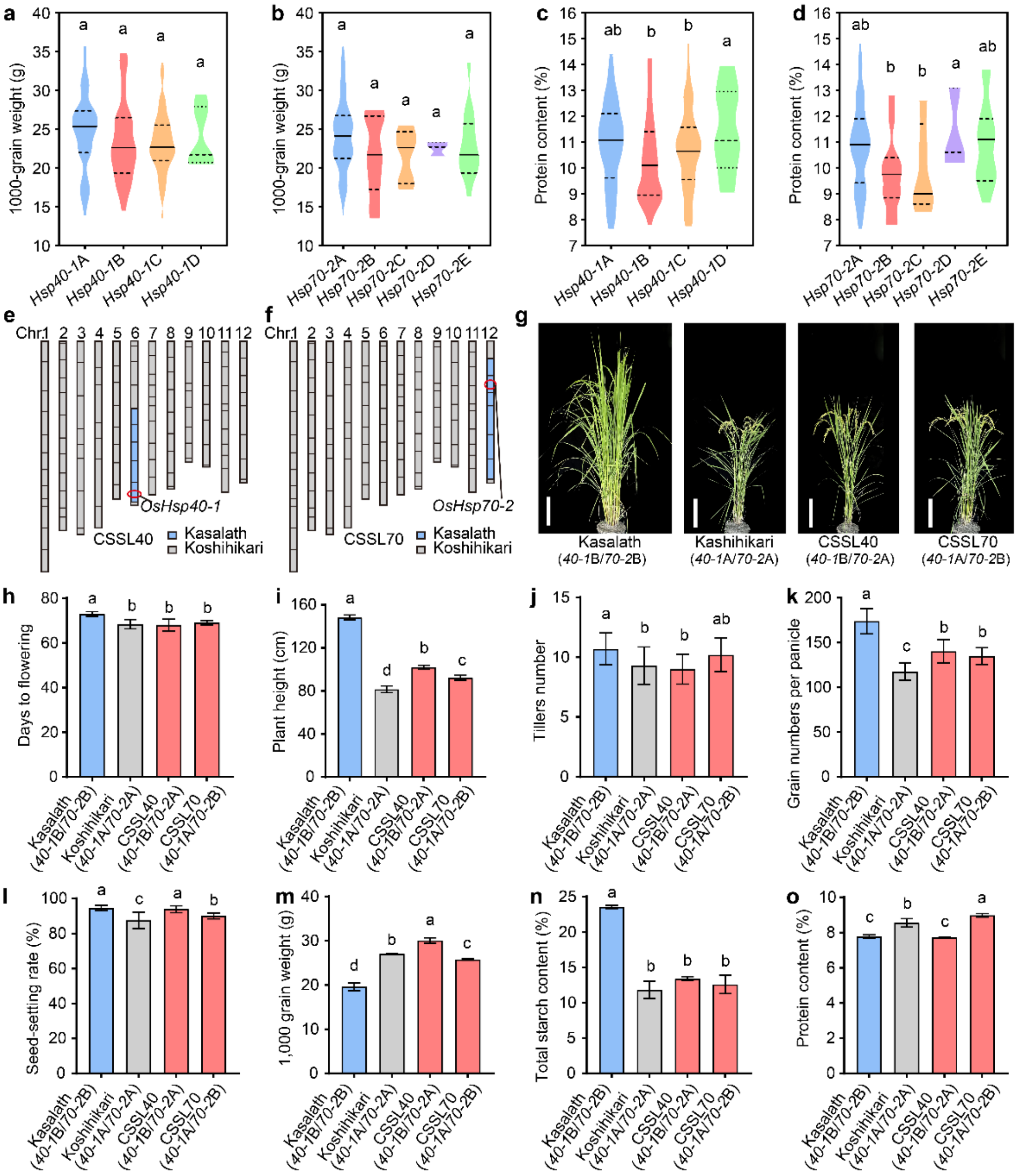

Supplementary Figure 16. The relationship between different variations of *OsHsp40-1*, *OsHsp70-2* and the important agronomic traits under nature high-temperature conditions

(a-d) The 1000 grain weight and protein content of rice accessions with the different types of *OsHsp40-1* (*Hsp40-1*A, B, C, and D) and *OsHsp70-2* (*Hsp70-2*A, B, C, D, and E) alleles under nature high-temperature conditions in Hangzhou, 2022 (2022HZ, daily average temperature 31.2 ℃ during filling stage). **(e-f)** Graphical genotypes of two chromosome segment substitution lines, CSSL40 and CSSL70, in which the chromosome segment containing *OsHsp40-1*A or *OsHsp70-2*A allele in Koshihikari (a *TEJ* accession) was substituted by Kasalath (an elite *AUS* rice) chromosome segment containing *OsHsp40-1*B or *OsHsp70-2*B, respectively. **(g)** The plants phenotypes of Kasalath, Koshihikari, CSSL40, and CSSL70 in 2022HZ, Scale bars, 10cm. **(h-m)** Days to flowering (h), plant height (i), tillers number (j), grain numbers per panicle (k), seed setting rate (l), and 1000 grain weight (m) of Kasalath, Koshihikari, CSSL40, and CSSL70 in 2022HZ. **(n-o)** The percent content of total starch (n) and protein (o) in endosperm of Kasalath, Koshihikari, CSSL40, and CSSL70 2022HZ. Data in (h-o) are means ± SD, n=10 in (h-l), =3 in (m-o), and no less than 200 grains per replication in (m). Different letters indicate significant difference at $p < 0.05$ by ANOVA and Duncan's test.

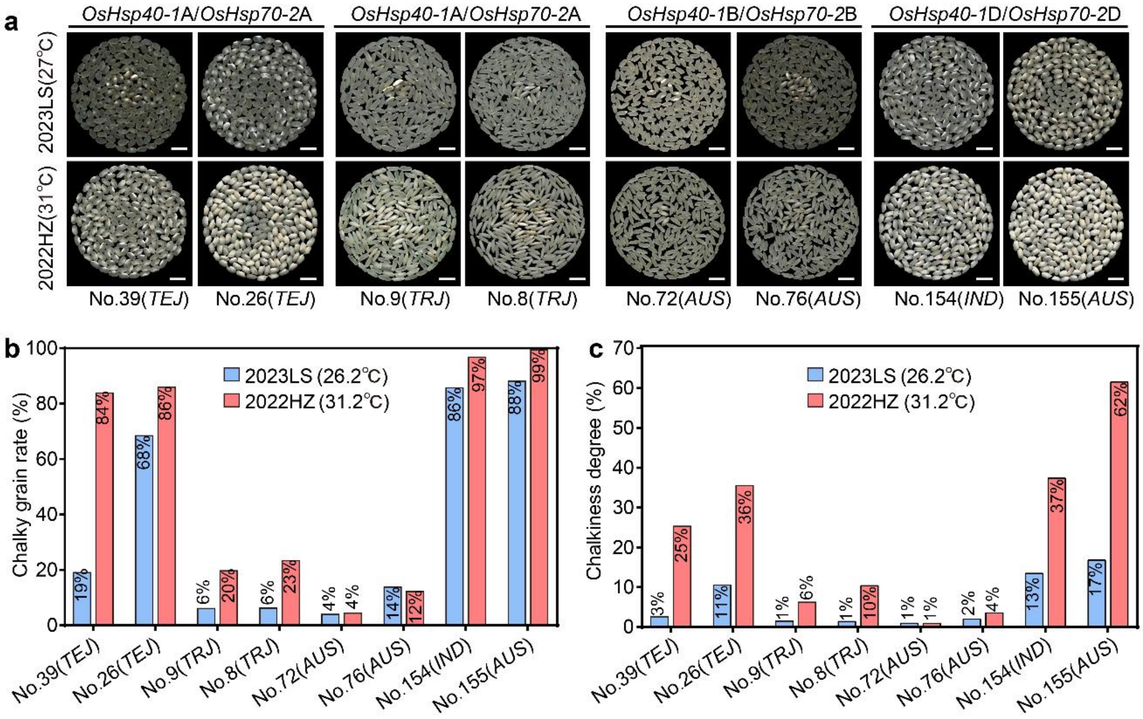

Supplementary Figure 17. The appearance of milled rice and chalky grain rate of some rice accessions with different combinations of *OsHsp40-1* and *OsHsp70-2* haplotypes.

(a) The appearance of milled rice of some rice accessions with different combinations of *OsHsp40-1* and *OsHsp70-2* haplotypes under field nature high-temperature conditions in Hangzhou, 2022 (2022HZ, daily average temperature 31.2 ℃ during filling stage) and field nature normal-temperature conditions in Lingshui, Hainan province, in spring 2023 (2023LS, daily average temperature 26.2 ℃ during filling stage). Scale bars, 10mm. **(b-c)** The chalky grain rate (b) and chalkiness degree (c) of some rice accessions with different combinations of *OsHsp40-1* and *OsHsp70-2* haplotypes under 2022HZ and 2023LS conditions.

Supplementary Tables:

Supplementary Table 1. The daily average temperature during the grain filling stage in Hangzhou, Zhejiang province, 2020-2023, and in Lingshui, Hainan province, 2023.

Hangzhou 2020		Hangzhou 2021		Hangzhou 2022		Hangzhou2023		Lingshui, 2023	
Date	Average temperature	Date	Average temperature	Date	Average temperature	Date	Average temperature	Date	Average temperature
2020/8/4	29℃	2021/8/3	27℃	2022/8/5	33℃	2023/8/3	30℃	2023/3/13	23℃
2020/8/5	30℃	2021/8/4	29℃	2022/8/6	34℃	2023/8/4	30℃	2023/3/14	23℃
2020/8/6	31℃	2021/8/5	28℃	2022/8/7	33℃	2023/8/5	32℃	2023/3/15	24℃
2020/8/7	31℃	2021/8/6	29℃	2022/8/8	34℃	2023/8/6	32℃	2023/3/16	23℃
2020/8/8	31℃	2021/8/7	28℃	2022/8/9	34℃	2023/8/7	30℃	2023/3/17	24℃
2020/8/9	31℃	2021/8/8	29℃	2022/8/10	34℃	2023/8/8	30℃	2023/3/18	25℃
2020/8/10	31℃	2021/8/9	30℃	2022/8/11	34℃	2023/8/9	30℃	2023/3/19	25℃
2020/8/11	31℃	2021/8/10	28℃	2022/8/12	34℃	2023/8/10	31℃	2023/3/20	25℃
2020/8/12	32℃	2021/8/11	28℃	2022/8/13	35℃	2023/8/11	31℃	2023/3/21	27℃
2020/8/13	32℃	2021/8/12	28℃	2022/8/14	35℃	2023/8/12	32℃	2023/3/22	28℃
2020/8/14	32℃	2021/8/13	26℃	2022/8/15	35℃	2023/8/13	32℃	2023/3/23	28℃
2020/8/15	32℃	2021/8/14	24℃	2022/8/16	33℃	2023/8/14	31℃	2023/3/24	28℃
2020/8/16	32℃	2021/8/15	25℃	2022/8/17	33℃	2023/8/15	29℃	2023/3/25	27℃
2020/8/17	31℃	2021/8/16	25℃	2022/8/18	33℃	2023/8/16	30℃	2023/3/26	27℃
2020/8/18	31℃	2021/8/17	26℃	2022/8/19	34℃	2023/8/17	30℃	2023/3/27	26℃
2020/8/19	31℃	2021/8/18	27℃	2022/8/20	33℃	2023/8/18	30℃	2023/3/28	27℃
2020/8/20	31℃	2021/8/19	28℃	2022/8/21	34℃	2023/8/19	29℃	2023/3/29	26℃
2020/8/21	31℃	2021/8/20	28℃	2022/8/22	35℃	2023/8/20	30℃	2023/3/30	27℃
2020/8/22	32℃	2021/8/21	28℃	2022/8/23	33℃	2023/8/21	30℃	2023/3/31	26℃
2020/8/23	31℃	2021/8/22	30℃	2022/8/24	29℃	2023/8/22	29℃	2023/4/1	28℃
2020/8/24	33℃	2021/8/23	31℃	2022/8/25	31℃	2023/8/23	29℃	2023/4/2	27℃
2020/8/25	33℃	2021/8/24	30℃	2022/8/26	29℃	2023/8/24	30℃	2023/4/3	27℃
2020/8/26	31℃	2021/8/25	29℃	2022/8/27	27℃	2023/8/25	29℃	2023/4/4	28℃
2020/8/27	29℃	2021/8/26	29℃	2022/8/28	26℃	2023/8/26	30℃	2023/4/5	29℃
2020/8/28	26℃	2021/8/27	29℃	2022/8/29	31℃	2023/8/27	31℃	2023/4/6	28℃
2020/8/29	27℃	2021/8/28	30℃	2022/8/30	28℃	2023/8/28	27℃	2023/4/7	28℃
2020/8/30	29℃	2021/8/29	30℃	2022/8/31	24℃	2023/8/29	23℃	2023/4/8	26℃
2020/8/31	30℃	2021/8/30	31℃	2022/9/1	26℃	2023/8/30	21℃	2023/4/9	25℃
2020/9/1	30℃	2021/8/31	30℃	2022/9/2	25℃	2023/8/31	23℃	2023/4/10	25℃
2020/9/2	28℃	2021/9/1	30℃	2022/9/3	23℃	2023/9/1	26℃	2023/4/11	26℃
2020/9/3	29℃	2021/9/2	29℃	2022/9/4	25℃	2023/9/2	27℃	2023/4/12	26℃
**Average value**	**30.0℃**	**Average value**	**28.4℃**	**Average value**	**31.2℃**	**Average value**	**29.2℃**	**Average value**	**26.2℃**

Supplementary Table 2. List of the interacting proteins screened in library of proteins of developing endosperm (10 DAF) by yeast two-hybrid assay using OsHsp70-2 as bait.

Gene	Protein
LOC_Os06g50870 (*OsHsp40-1*)	DNL zinc finger domain containing protein, putative, expressed
LOC_Os05g33570 (*OsPPDKB*)	phosphatidic acid phosphatase-related, putative, expressed
LOC_Os11g33000 (*OsSSA5*)	SSA5-2S albumin seed storage family protein precursor, expressed
LOC_Os06g06790 (*OsPDIL1-5*)	OsPDIL1-5 protein disulfide isomerase PDIL1-5, expressed
LOC_Os01g67860 (*OsFBA*)	fructose-bisphospate aldolase isozyme, putative, expressed
LOC_Os07g38240 (*OsSAP16*)	ZOS7-05-C2H2 zinc finger protein, expressed
LOC_Os03g46100 (*OsCUP*)	cupin domain containing protein, expressed
LOC_Os12g27830 (*OsDEH*)	dehydrogenase/reductase, putative, expressed
LOC_Os02g10660 (*OsGLY*)	glycosyl hydrolases family 17, putative, expressed
LOC_Os10g41870 (*OsCAO1*)	chlorophyllide a oxygenase, chloroplast putative, expressed

Supplementary Table 3. The haplotypes of *OsHsp40-1* and *OsHsp70-2*, and grain appearance quality of 155 rice accessions.

Number	Accession Name	Ecotype	*OsHsp40-1* haplotype	*OsHsp70-2* haplotype	Origin	Chalky Ratio (%)	Chalkiness Degree (%)
1	81A32	*TRJ*	TypeA	TypeA	CHINA	78	35
2	CALIFORNIABELLE	*TRJ*	TypeA	TypeA	UNITEDSTATES	75	27
3	80050YR72136-36	*TRJ*	TypeA	TypeA	AUSTRALIA	34	13
4	PELDE	*TRJ*	TypeA	TypeA	NAMIBIA	21	9
5	MAYBELLE	*TRJ*	TypeA	TypeA	UNITEDSTATES	30	14
6	B6311A5553-16-2	*TRJ*	TypeA	TypeA	UNITEDSTATES	10	4
7	IR71525-19-1-1	*TRJ*	TypeA	TypeA	NAMIBIA	67	30
8	JACINTO	*TRJ*	TypeA	TypeA	NAMIBIA	23	10
9	IR63380-16	*TRJ*	TypeA	TypeA	NAMIBIA	20	6
10	PLUS	*TRJ*	TypeA	TypeA	NAMIBIA	52	25
11	BOND	*TRJ*	TypeA	TypeA	UNITEDSTATES	24	6
12	TEBONNET	*TRJ*	TypeA	TypeA	UNITEDSTATES	28	10
13	WAB56-125	*TRJ*	TypeA	TypeA	WAB56-125	34	9
14	KATY	*TRJ*	TypeA	TypeA	UNITEDSTATES	17	6
15	79UPLA	*TRJ*	TypeA	TypeA	NAMIBIA	32	13
16	SKYBONNET	*TRJ*	TypeA	TypeA	UNITEDSTATES	18	5
17	91UPLA	*TRJ*	TypeA	TypeA	NAMIBIA	19	6
18	SINAMPUENG	*TEJ*	TypeA	TypeA	THAILAND	97	57
19	ALEXANDROS	*TRJ*	TypeA	TypeA	NAMIBIA	48	25
20	KHUDWANIACC202	*TRJ*	TypeA	TypeA	INDIA	10	3
21	INIATACUARI	*TRJ*	TypeA	TypeA	NAMIBIA	22	5
22	BETIS	*TEJ*	TypeA	TypeA	SPAIN	40	18
23	CHUNGYI	*TEJ*	TypeA	TypeA	CHINA	100	57
24	CALENDAL	*TEJ*	TypeA	TypeA	NAMIBIA	98	60
25	CT58	*TEJ*	TypeA	TypeA	NAMIBIA	63	45
26	S201	*TEJ*	TypeA	TypeA	UNITEDSTATES	86	36
27	S102/2	*TEJ*	TypeA	TypeA	NAMIBIA	86	43
28	YUNLEN13	*TEJ*	TypeA	TypeA	CHINA	79	45
29	SUPER	*TEJ*	TypeA	TypeA	NAMIBIA	97	64
30	LIGEN2	*TEJ*	TypeA	TypeA	CHINA	86	56
31	JUBILIENI	*TEJ*	TypeA	TypeA	NAMIBIA	17	10
32	CHALBYEO	*TEJ*	TypeA	TypeA	SOUTHKOREA	96	46
33	TAICHUNG65	*TEJ*	TypeA	TypeA	TAIWAN	19	4
34	RUBI	*TEJ*	TypeA	TypeA	NAMIBIA	7	2
35	ZHENG-DAO5	*TEJ*	TypeA	TypeA	CHINA-HENAN	63	19
36	NABATEDASMAR	*TEJ*	TypeA	TypeA	EGYPT	62	16
37	HONG-QI5	*TEJ*	TypeA	TypeA	CHINA-HUNAN	99	43
38	DANYANNUO	*TEJ*	TypeA	TypeA	CHINA	8	1
39	HAO-BA-YONG1	*TEJ*	TypeA	TypeA	CHINA-YUNNAN	84	25
40	ZAO-SHU-NONG-HU6B	*TEJ*	TypeA	TypeA	CHINA-HUNAN	51	17
41	MUNJI	*IND*	TypeA	TypeA	PAKISTAN	48	12
42	P335	*ARO*	TypeA	TypeA	LIBERIA	95	60
43	TAIPEI167	*ARO*	TypeA	TypeA	TAIWAN	59	20

44	CT45	*TEJ*	TypeA	TypeA	INDIA	71	23
45	SAKHA103	*TEJ*	TypeA	TypeE	NAMIBIA	57	21
46	WUYUNGENG3	*TEJ*	TypeA	TypeE	CHINA-JIANGSU	53	17
47	LI-XIN-KENG	*TEJ*	TypeA	TypeE	SICHUAN	72	18
48	XIAN-GAIB	*IND*	TypeA	TypeE	CHINA-JIANGXI	93	28
49	MANSARADHAN	*AUS*	TypeB	TypeA	NEPAL	9	3
50	KAMULI	*AUS*	TypeB	TypeA	NEPAL	7	2
51	CHAODO	*IND*	TypeB	TypeA	LAOS	80	40
52	RP1153-20-14	*IND*	TypeB	TypeA	INDIA	76	31
53	YANONGZAO4	*IND*	TypeB	TypeA	CHINA	84	29
54	IR2344-P1PB-9-3-2B	*IND*	TypeB	TypeA	PHILIPPINES	32	13
55	71011	*IND*	TypeB	TypeA	AUSTRALIA	51	21
56	CHISHENGTAO	*IND*	TypeB	TypeA	CHINA	65	17
57	QINGTAIAI	*IND*	TypeB	TypeA	CHINA	23	8
58	SANHUANGZHANNO2	*IND*	TypeB	TypeA	NAMIBIA	13	3
59	IR65483-111-5-9-2-11	*IND*	TypeB	TypeA	PHILIPPINES	38	11
60	EDAKKADAN0-69-27	*IND*	TypeB	TypeA	INDIA	18	3
61	DANG-YU5	*IND*	TypeB	TypeA	CHINA-ANHUI	15	4
62	104UPLA	*TRJ*	TypeB	TypeA	NAMIBIA	39	16
63	32UPLA	*TRJ*	TypeB	TypeA	NAMIBIA	49	24
64	IRAT104	*TRJ*	TypeB	TypeA	NAMIBIA	38	11
65	62667	*TRJ*	TypeB	TypeA	NAMIBIA	39	11
66	MARANHAOBRANCO	*TRJ*	TypeB	TypeA	BRAZIL	21	6
67	IRAT 112	*TRJ*	TypeB	TypeA	IRAT 112	34	14
68	YR83-23-11	*TRJ*	TypeB	TypeA	AUSTRALIA	15	3
69	AUS278	*AUS*	TypeB	TypeB	BANGLADESH	15	5
70	ARC14756	*AUS*	TypeB	TypeB	INDIA	53	19
71	KANGRI	*AUS*	TypeB	TypeB	PAKISTAN	46	15
72	SONAAUS	*AUS*	TypeB	TypeB	INDIA	4	1
73	UPRH166	*AUS*	TypeB	TypeB	INDIA	13	5
74	T21	*AUS*	TypeB	TypeB	INDIA	11	3
75	UPRH58	*AUS*	TypeB	TypeB	INDIA	8	1
76	DEVARASI	*AUS*	TypeB	TypeB	NEPAL	19	4
77	LALSAR	*AUS*	TypeB	TypeB	NEPAL	28	7
78	DODGUI	*AUS*	TypeB	TypeC	INDIA	51	13
79	AUS439	*AUS*	TypeB	TypeC	BANGLADESH	12	4
80	BERENJ1277S	*TRJ*	TypeB	TypeC	AFGHANISTAN	30	10
81	AIJIAOZI	*IND*	TypeB	TypeD	CHINA	42	15
82	JINJUNDAO	*IND*	TypeB	TypeD	CHINA	46	19
83	DONG-TING-WAN-XIAN	*IND*	TypeB	TypeD	CHINA-HUBEI	39	12
84	KANAIBASHI	*AUS*	TypeB	TypeE	INDIA	18	6
85	BEGUM	*AUS*	TypeB	TypeE	INDIA	37	8
86	KANPURI	*AUS*	TypeB	TypeE	INDIA	44	15
87	MOTIA	*AUS*	TypeB	TypeE	PAKISTAN	31	9
88	E2024	*IND*	TypeC	TypeA	CHINA	12	8

89	NANJINGXIAODAO	*IND*	TypeC	TypeA	CHINA	84	45
90	JIANG-NONG-ZAO1B	*IND*	TypeC	TypeA	CHINA-JIANGXI	44	16
91	JING-HUB	*IND*	TypeC	TypeA	CHINA-ANHUI	69	28
92	IRGA318-11-6-9-2B	*IND*	TypeC	TypeA	COLOMBIA	13	5
93	IRGA370-42-1-1F-C-1	*IND*	TypeC	TypeA	COLOMBIA	23	6
94	E4197	*IND*	TypeC	TypeA	CHINA	27	8
95	IR3839-1	*IND*	TypeC	TypeA	PHILIPPINES	52	16
96	CAUVERY	*IND*	TypeC	TypeA	INDIA	62	20
97	IR77298-14-1-2-10	*IND*	TypeC	TypeA	NAMIBIA	22	7
98	WAS170-B-B-1-1	*IND*	TypeC	TypeA	NAMIBIA	58	34
99	WAS173-B-B-6-2-2	*IND*	TypeC	TypeA	NAMIBIA	61	28
100	IRI339	*IND*	TypeC	TypeA	SOUTHKOREA	81	28
101	ICTACRISPO38	*IND*	TypeC	TypeA	GUATEMALA	34	11
102	LIU-YE-NIAN	*IND*	TypeC	TypeA	CHINA-HUBEI	33	19
103	IR57920-AC25-2-B	*IND*	TypeC	TypeA	NAMIBIA	15	5
104	B6136E3-TB-0-1-5	*IND*	TypeC	TypeA	INDONESIA	48	14
105	DAGUAI7	*IND*	TypeC	TypeA	CHINA	80	31
106	SHANGU	*IND*	TypeC	TypeA	CHINA	77	27
107	TESANAI2	*IND*	TypeC	TypeA	CHINA	57	11
108	GUI-CHAO 2	*IND*	TypeC	TypeA	CHINA-GUANGDONG	81	25
109	SAMBALAMALO	*IND*	TypeC	TypeA	NAMIBIA	23	8
110	AUS177	*IND*	TypeC	TypeA	BANGLADESH	12	4
111	EXMARABAGURUKU	*IND*	TypeC	TypeA	NIGERIA	13	6
112	KHAONAHNGPRUNG	*IND*	TypeC	TypeA	THAILAND	27	5
113	TAICHUNGWOOGEN2	*IND*	TypeC	TypeA	TAIWAN	36	8
114	K24	*IND*	TypeC	TypeA	UGANDA	30	9
115	XU-GU-NUO	*IND*	TypeC	TypeA	CHINA-HUNAN	79	36
116	XIANG-WAN-XIAN1	*IND*	TypeC	TypeA	CHINA-HUNAN	33	9
117	IRGA659-1-2-2-2	*IND*	TypeC	TypeA	COLOMBIA	49	28
118	CICA 9	*IND*	TypeC	TypeA	CICA 6	61	17
119	INIAP10	*IND*	TypeC	TypeA	ECUADOR	26	9
120	B3913B16-20ST28	*IND*	TypeC	TypeA	INDONESIA	28	6
121	WAS198-B-3-1-3	*IND*	TypeC	TypeA	NAMIBIA	17	5
122	KHAOGRADOOKCHAHNG	*IND*	TypeC	TypeA	THAILAND	27	7
123	ARAURE1	*IND*	TypeC	TypeA	VENEZUELA	19	6
124	KAHATAWEE	*IND*	TypeC	TypeA	SRILANKA	59	12
125	BAO-XIE123B	*IND*	TypeC	TypeA	CHINA-HUNAN	16	5
126	AMISTAD82	*IND*	TypeC	TypeA	CUBA	2	1
127	IRGA411-1-6-1F-A	*IND*	TypeC	TypeA	COLOMBIA	12	3
128	UPR1201-1-20-1	*IND*	TypeC	TypeA	INDIA	21	3
129	UPLRI7	*IND*	TypeC	TypeA	NAMIBIA	33	9
130	WAS63-22-5-9-10-1	*IND*	TypeC	TypeA	NAMIBIA	21	4
131	BINUHANGIN	*IND*	TypeC	TypeA	PHILIPPINES	44	12
132	ITA117	*IND*	TypeC	TypeA	NIGERIA	59	14
133	PORONG	*IND*	TypeC	TypeA	INDONESIA	34	10

134	B6144-MR-6-0-0	*IND*	TypeC	TypeA	NAMIBIA	62	11
135	CIRAD403	*TRJ*	TypeC	TypeA	NAMIBIA	55	26
136	BINARITOS	*TRJ*	TypeC	TypeA	PHILIPPINES	15	5
137	YOSHINOMOCHI	*TRJ*	TypeC	TypeA	JAPAN	85	30
138	BA-WANG-BIAN	*TEJ*	TypeC	TypeA	CHINA-HUBEI	64	17
140	NING-HUI21	*TEJ*	TypeC	TypeA	CHINA-JIANGSU	67	32
141	JHONA101	*AUS*	TypeC	TypeB	PAKISTAN	18	8
142	GENIT	*TEJ*	TypeC	TypeB	ARGENTINA	64	19
143	JADO	*ARO*	TypeC	TypeC	NEPAL	59	24
144	FRAGANCE	*TRJ*	TypeC	TypeC	NAMIBIA	22	7
145	CNA4081	*IND*	TypeC	TypeE	NAMIBIA	85	42
146	AUS449	*AUS*	TypeC	TypeE	BANGLADESH	48	18
147	TILOKCHAN	*AUS*	TypeC	TypeE	BANGLADESH	38	9
148	SLO19	*AUS*	TypeC	TypeE	INDIA	68	32
149	QI-TOU-BAI-GU	*IND*	TypeD	TypeA	CHINA-YUNNAN	99	47
150	CISOKAN	*IND*	TypeD	TypeA	INDONESIA	73	30
151	CHATAO	*TRJ*	TypeD	TypeA	BRAZIL	98	55
152	80A97YR303-304-1-4	*TRJ*	TypeD	TypeA	AUSTRALIA	35	11
153	ZHENSHAN97B	*IND*	TypeD	TypeA	SICHUAN	99	50
154	ZHONG-NONG4	*IND*	TypeD	TypeE	SICHUAN	97	37
155	KALABOKRI	*AUS*	TypeD	TypeE	BANGLADESH	99	62

Supplementary Table 4. Primers used in this study.

Name	Forward (5'-3')	Reverse (5'-3')
**Primers for map-based cloning**		
SSR12-5	CACAAAGGCGTGTGGGTTAG	GAGTCACGGGATGTTGCC
SSR12-26	AGAGAGCCCCTAAATTTCCG	AGGTACGCTCACCTGTGGAC
RM28179	GGTTCGAGGTGATCACTGCTTCC	GTGGTCACCGAGGTGGTTGAGG
RM28033	CAGCTTATCAGGTCATGGTCAGG	GTGGCAACTTAGTAGCGTTTGTAGG
RM27877	GGAAGCCATGAAAGATGTGTTGC	AATTTCTCCGAGCACCTGAAACG
RM27808	GGAAGTGCCCGATTAGTATAGG	ATCACCTACTACCTCCATTTCAGG
In1	TGCAAGACACCAGAAAACTGA	TATTCCGAACACAGCATGGA
In2	GCTCTCATCCATCCATAGCAAGC	CCTGCGAGATGATATTCAGATGC
In3	GTCATCGTGAGGCCCATTAAGC	GGAAGAAGAAGACGTCCTGTAGCC
**Primers for vector construction**		
2300-Hsp70-2	CCATGATTACGAATTCACGGTCATTTGTCAGGTCAT	CGACTCTAGAGGATCCAGGGATCCAAAGGCTGATACT
1390-Hsp70-2	TTACTTCTGCACTAGGTACCATGGCCTCCTTCACCTCCCA	GAATTCCCGGGGATCCTCAATTGCTGTCAGTGAAAT
1390-Hsp40-1	TTACTTCTGCACTAGGTACCATGGAGTCCGTCGCGATCG	GAATTCCCGGGGATCCTCACAGAATTGGAAATATG
1305ubi- Hsp40-1-GFP	GGTACCTGCAACTAGTATGGAGTCCGTCGCGATCGC	TGCTCACCATGGATCCCAGAATTGGAAATATGTTGT
1305ubi-Hsp70-2	CTAGGTACCTGCAACTAGTATGGCCTCCTTCACCTCCCA	CCTTGCTCACCATGGATCCATTGCTGTCAGTGAAAT
pAN580- Hsp40-1	GCCCAGATCAACTAGTATGGAGTCCGTCGCGATCGC	TCGAGACGTCTCTAGACAGAATTGGAAATATGTTGT
pAN580-Hsp70-2-GFP	GCCCAGATCAACTAGTATGGCCTCCTTCACCTCCCA	TCGAGACGTCTCTAGAATTGCTGTCAGTGAAAT
pAN580-GBSSI-GFP	GCCCAGATCAACTAGTATGTCGGCTCTCACCACG	TCGAGACGTCTCTAGAAGGAGCAGCCACGTTCTC
pAN580-PPDKB-GFP	GCCCAGATCAACTAGTATGCCGTCGGTTTCGAGGGCC	TCGAGACGTCTCTAGAGAGGAGCACCTGAGCTGC
pA7- Hsp40-1-CFP	GCAGCCCGGGGGATCCATGGAGTCCGTCGCGATCGC	TGGCGGCCGCTCTAGACAGAATTGGAAATATGTTGT
pA7-GBSSI-CFP	GCAGCCCGGGGGATCCATGTCGGCTCTCACCACG	TGGCGGCCGCTCTAGAAGGAGCAGCCACGTTCTC
pA7-PPDKB-CFP	GCAGCCCGGGGGATCCATGCCGTCGGTTTCGAGGGCC	TGGCGGCCGCTCTAGAGAGGAGCACCTGAGCTGC
pGBT9-Hsp70-2	TGTATCGCCGGAATTCATGGCCTCCTTCACCTCCCA	GCAGGTCGACGGATCCTCAATTGCTGTCAGTGAAAT
pGADT7-Hsp70-2	GGAGGCCAGTGAATTCATGGCCTCCTTCACCTCCCA	CGAGCTCGATGGATCCTCAATTGCTGTCAGTGAAAT
pGBT9-N-Hsp70-2	TGTATCGCCGGAATTCATGGCCTCCTTCACCTCCCA	GCAGGTCGACGGATCCCTTATCGAAGTCATCGCCAC
pGBT9-C-Hsp70-2	TGTATCGCCGGAATTCAAAGTTGTGGATTGGCTTGC	GCAGGTCGACGGATCCTCAATTGCTGTCAGTGAAAT
pGBT9-Hsp40-1	TGTATCGCCGGAATTCATGGAGTCCGTCGCGATCGCC	GCAGGTCGACGGATCCTCACAGAATTGGAAATATGT
pGADT7-Hsp40-1	GGAGGCCAGTGAATTCATGGAGTCCGTCGCGATCGC	CGAGCTCGATGGATCCTCACAGAATTGGAAATATGT
pGBT9-N-Hsp40-1	TGTATCGCCGGAATTCATGGAGTCCGTCGCGATCGC	GCAGGTCGACGGATCCCATCCGCCGCCGTGGGGACT
pGBT9-C-Hsp40-1	TGTATCGCCGGAATTCCGCGTGGCCTTCACCTGCAA	GCAGGTCGACGGATCCTCCTTCGTAGCGGAAGTCTG
pGADT7-N-Hsp40-1	GGAGGCCAGTGAATTCATGGAGTCCGTCGCGATCGC	CGAGCTCGATGGATCCCATCCGCCGCCGTGGGGACT

pGADT7-C-Hsp40-1	GGAGGCCAGTGAATTCCGCGTGGCC TTCACCTGCAA	CGAGCTCGATGGATCCTCCTTCGTAGCGGA AGTCTG
pGADT7-GBSSI	GGAGGCCAGTGAATTCATGTCGGCT CTCACCACG	CGAGCTCGATGGATCCTCAAGGAGCAGCC ACGTT
pGADT7-PPDKB	GGAGGCCAGTGAATTCATGGCGCCG GCCGCTCATCGG	CGAGCTCGATGGATCCTCACAGCATCACCT GCGCTGC
2300S-YC-Hsp40-1	AGGAGCTCGGTACCCGGGATGGAGT CCGTCGCGATCGC	ATGGGTACATACTAGTCAGAATTGGAAAT ATGTTGT
1300S-YN-Hsp70-2	GAGGAGGATCTTCCCGGGATGGCCT CCTTCACCTCCCAG	ATGCCTGCAGGTCGACATTGCTGTCAGTGA AATCCGC
2300S-YC-Hsp70-2	AGGAGCTCGGTACCCGGGATGGCCT CCTTCACCTCCCAG	ATGGGTACATACTAGTATTGCTGTCAGTGA AATCCGC
1300S-YN-Hsp40-1	GAGGAGGATCTTCCCGGGATGGAGT CCGTCGCGATCGC	ATGCCTGCAGGTCGACCAGAATTGGAAAT ATGTTGT
2300S-YC-GBSSI	AGGAGCTCGGTACCCGGGATGTCGG CTCTCACCACG	ATGGGTACATACTAGTTCAAGGAGCAGCC ACGTT
2300S-YC-PPDKB	AGGAGCTCGGTACCCGGGATGCCGT CGGTTTCGAGG	ATGGGTACATACTAGTGAGGAGCACCTGA GCTGC
1300nLUC-Hsp40-1	ACGGGGGACGAGCTCGGTACCATGG AGTCCGTCGCGATC	CGCGTACGAGATCTGGTCGACCAGAATTG GAAATATGTT
1300cLUC-Hsp70-2	TACGCGTCCCGGGGCGGTACCATGG CCTCCTTCACCTCC	ATACGAACGAAAGCTCTGCAGTCAATTGCT GTCAGTGAA
1300cLUC-GBSSI	TACGCGTCCCGGGGCGGTACCATGT CGGCTCTCACCACG	ATACGAACGAAAGCTCTGCAGTCAAGGAG CAGCCACGTT
1300cLUC-PPDKB	TACGCGTCCCGGGGCGGTACCATGC CGTCGGTTTCGAGG	ATACGAACGAAAGCTCTGCAGTCAGAGGA GCACCTGAGC
pET28a-Hsp40-1	GGTGGTGGTGCTCGAGATGGAGTCC GTCGCGATCGCC	TCGCGGATCCGAATTCCAGAATTGGAAAT ATGTTGTC
pGEX4T-1-Hsp70-2	GGTTCCGCGTGGATCCATGGCCTCCT TCACCTCCCA	GTCGACCCGGGAATTCATTGCTGTCAGTGA AATCCG
pGEX4T-1-Hsp40-1	GGTTCCGCGTGGATCCATGGAGTCC GTCGCGATCGC	GTCGACCCGGGAATTCCAGAATTGGAAAT ATGTTGT
pGEX4T-1-N-Hsp40-1	GGTTCCGCGTGGATCCATGGAGTCC GTCGCGATCGC	GTCGACCCGGGAATTCCATCCGCCGCCGTG GGGACT
pGEX4T-1-C-Hsp40-1	GGTTCCGCGTGGATCCCGCGTGGCC TTCACCTGCAA	GTCGACCCGGGAATTCTCCTTCGTAGCGGA AGTCTG
pGEX4T-1-N-Hsp70-2	GGTTCCGCGTGGATCCATGGCCTCCT TCACCTCCCA	GTCGACCCGGGAATTCCTTATCGAAGTCAT CGCCAC
pGEX4T-1-C-Hsp70-2	GGTTCCGCGTGGATCCAAAGTTGTG GATTGGCTTGC	GTCGACCCGGGAATTCATTGCTGTCAGTGA AA
pET28a-PPDKB	GGTGGTGGTGCTCGAGATGCCGTCG GTTTCGAGGGC	TCGCGGATCCGAATTCGAGGAGCACCTGA GCTGCA
pET28a-GBSSI	GGTGGTGGTGCTCGAGATGTCGGCT CTCACCACGT	TCGCGGATCCGAATTCAGGAGCAGCCACG TTCTC
pHY35S(3xFlag)-Hsp40-1	GAGGACAATTGGGTACCATGGAGTC CGTCGCGATC	TCGATGGATCCGTCGACCAGAATTGGAAA TATGTT
pYBA1132eGFP-Hsp70-2	CTAGAACTAGTGGATCCATGGCCTC CTTCACCTCC	AGCTTGATATCGAATTCATTGCTGTCAGTG AAATC
pYBA1132eGFP-Hsp40-1	CTAGAACTAGTGGATCCATGGAGTC CGTCGCGATC	AGCTTGATATCGAATTCCAGAATTGGAAAT ATGTT
pHY35S(3xFlag)-PPDKB	GAGGACAATTGGGTACCATGCCGTC GGTTTCGAGG	TCGATGGATCCGTCGACGAGGAGCACCTG AGCTGC
pHY35S(3xFlag)-GBSSI	GAGGACAATTGGGTACCATGTCGGC TCTCACCACG	TCGATGGATCCGTCGACAGGAGCAGCCAC GTTCT
**Primers for CRISPR creation and mutant identification**		
OsHsp70-2cas9	CAGAAGGTGGTGGGGATCGACCT	AACAGGTCGATCCCCACCACCTT
OsHsp40-1cas9	CAGTGGAGAGGCCGTACGAGGCG	AACCGCCTCGTACGGCCTCTCCA

OsHsp70-2T	AACTGCTACTGCTGATGGGC	TGAGGACAAGAAAGTGACCTGT
OsHsp40-1T	AAGTTCCGTGTCGTGCTGG	AGATGACAACGTCAGCAGCAT
**Primers for sequencing**		
OsHsp40-1-hap-1	AAGTTCCGTGTCGTGCTGG	GGGAGTGAGGGTTCGTATCG
OsHsp40-1-hap-2	AAGTTCCGTGTCGTGCTGG	GGAGGGAGTGAGGGTTCGT
OsHsp70-2-hap-1	CTCCGCCTGAATCCTTCTCCC	ACCACCTTCTCGCACGCCAC
OsHsp70-2-hap-2	GAGAAAGCGAAGATGGAA	CAGTAGCAGTTATGAAAGG
OsHsp70-2-hap-3	GATAAATGTTCTTCAGGGAG	GTGGAAAAGAAAATCTACAA
OsHsp70-2-hap-4	GCGAAGATACAAATGCCTAC	TGAGCTTTGCATCCACCT
**Primers for real time PCR**		
Hsp70-2-RT	GCCTAATGCTGGTGCTGCTG	GTCCCTTACCGCCTGAGCTT
Hsp40-1-RT	TACACTGACGGCACTGTCTT	TCTCCTTCGTAGCGGAAGTC
PPDKB-RT	CATGCACTGTTCGAGGAGAA	GGGAAATGGCTCTCCCTTAG
GBSSI-RT	TCCGAGAGGTTCAGGTCATC	ATGAGCTCCTCGGCGTAGTA
Actin1-RT	CCCTCCTGAAAGGAAGTACAGTGT	GTCCGAAGAATTAGAAGCATTTCC

456
